# Optimising a person-centred approach to stopping medicines in older people with multimorbidity and polypharmacy using the DExTruS framework: a realist review

**DOI:** 10.1186/s12916-022-02475-1

**Published:** 2022-08-31

**Authors:** Amadea Turk, Geoffrey Wong, Kamal R. Mahtani, Michelle Madden, Ruaraidh Hill, Ed Ranson, Emma Wallace, Janet Krska, Dee Mangin, Richard Byng, Daniel Lasserson, Joanne Reeve

**Affiliations:** 1 Nuffield Department of Primary Care Health Sciences, Radcliffe Observatory Quarter, University of Oxford, Oxford, OX2 6GG UK; 2 Liverpool Reviews & Implementation Group, Institute of Population Health, University of Liverpool, Liverpool, L69 3BX UK; 3 Academy of Primary Care, Hull York Medical School, Allam Medical Building, University of Hull, Hull, HU6 7RX UK; 4 Department of General Practice RCSI University of Medicine and Health Sciences, Dublin 2, Ireland; 5 Medway School of Pharmacy, Universities of Greenwich and Kent, Chatham Maritime, Kent, ME4 4TB UK; 6 Department of Family Medicine, McMaster University, Hamilton, ON L8P 1H6 Canada; 7 Community and Primary Care Research Group, Peninsula Medical School, University of Plymouth, Plymouth, PL4 8AA UK; 8 Health Sciences, Warwick Medical School, University of Warwick, Coventry, CV4 7AL UK

**Keywords:** Realist review, Evidence synthesis, Deprescribing, Polypharmacy, Person-centred care

## Abstract

**Background:**

Tackling problematic polypharmacy requires tailoring the use of medicines to individual circumstances and may involve the process of deprescribing. Deprescribing can cause anxiety and concern for clinicians and patients. Tailoring medication decisions often entails beyond protocol decision-making, a complex process involving emotional and cognitive work for healthcare professionals and patients. We undertook realist review to highlight and understand the interactions between different factors involved in deprescribing and to develop a final programme theory that identifies and explains components of good practice that support a person-centred approach to deprescribing in older patients with multimorbidity and polypharmacy.

**Methods:**

The realist approach involves identifying underlying causal mechanisms and exploring how, and under what conditions they work. We conducted a search of electronic databases which were supplemented by citation checking and consultation with stakeholders to identify other key documents. The review followed the key steps outlined by Pawson et al. and followed the RAMESES standards for realist syntheses.

**Results:**

We included 119 included documents from which data were extracted to produce context-mechanism-outcome configurations (CMOCs) and a final programme theory. Our programme theory recognises that deprescribing is a complex intervention influenced by a multitude of factors. The components of our final programme theory include the following: a supportive infrastructure that provides clear guidance around professional responsibilities and that enables multidisciplinary working and continuity of care, consistent access to high-quality relevant patient contextual data, the need to support the creation of a shared explanation and understanding of the meaning and purpose of medicines and a trial and learn approach that provides space for monitoring and continuity. These components may support the development of trust which may be key to managing the uncertainty and in turn optimise outcomes. These components are summarised in the novel DExTruS framework.

**Conclusion:**

Our findings recognise the complex interpretive practice and decision-making involved in medication management and identify key components needed to support best practice. Our findings have implications for how we design medication review consultations, professional training and for patient records/data management. Our review also highlights the role that trust plays both as a central element of tailored prescribing and a potential outcome of good practice in this area.

**Supplementary Information:**

The online version contains supplementary material available at 10.1186/s12916-022-02475-1.

## Background

Polypharmacy, the concurrent use of multiple medicines in a single person, is common practice in modern healthcare, with an estimated 1 in 5 patients taking five or more medicines a day [1]. Polypharmacy can be an important part of a patient’s treatment plan, extending life expectancy and improving quality of life [1]. Problematic polypharmacy occurs when the use of multiple medications on a long-term basis does not achieve the intended benefits or when the potential risks outweigh the intended benefits [1]. Problematic polypharmacy is associated with treatment burden (40% of people with polypharmacy report feeling significantly burdened by their medication [2]), potential harm and waste (through non-concordance) [1] and thus presents a significant challenge for healthcare services, healthcare professionals and patients.

Deprescribing, the process of supervised withdrawal or dose reduction of potentially inappropriate medicines [3], has been highlighted as an important strategy for tackling problematic polypharmacy [1]. However, deprescribing long-term medicines is a process that can cause anxiety and concern for both healthcare professionals and patients [4, 5]. Patients may be worried about losing the benefits they believe their medicines confer them [6–8] and the uncertainties around the management of their condition. Healthcare professionals may be concerned about the safety and uncertain impact of stopping medicines [3, 5] as well as the challenges of managing the process of withdrawal [9].

Guidelines on best practice have been criticised for having a single disease focus and not acknowledging the needs of patients with multimorbidity [10] and for not considering the evidence relating to patients’ lived experiences [11]. While there are a number of tools such as the Beers Criteria [12] and the STOPP/START tool [13] that help identify potentially inappropriate drugs and consider dose and duration, these tools do not provide detailed practical guidance on how to navigate uncertainties and how to achieve deprescribing in practice.

A King’s Fund report on the challenges of polypharmacy underscored the importance of adopting a person-centred and tailored approach to medication management, which acknowledges the perspectives of the patient and their families and carers [1, 14]. The report highlighted that it is important to recognise that the perspectives and priorities of patients may not align with the priorities of the prescriber [1, 14]. In such situations, “compromises may be needed” ([1], p. 32) between the goal to optimise the medication a patient should be on based on current guidelines, and patient goals and preferences based on their individual circumstances [1]. Tailored or person-centred deprescribing can therefore involve complex decision-making and may require prescribers to make beyond-guideline decisions.

Studies of barriers to delivering tailored person-centred prescribing reveal a number of challenges which may not be addressed by current guidelines for medicines optimisation [4, 15]. Healthcare professionals report anxiety and a lack of confidence undertaking tailored prescribing due to a lack of support in four key areas across the system and policy level [4, 16]. The barriers include having permission to work beyond guidelines and existing frameworks, the lack of space within their workload needed to prioritise and undertake tailored deprescribing, a lack of skills in complex decision making and the confidence to use them and performance management processes [4].

Tailored deprescribing may be described as a “wicked problem”—a set of complex problems that cannot be addressed because of incomplete, competing and changing requirements, but that can be managed through iterative and adaptive responses [17, 18]. In recognising that tailored deprescribing is a complex intervention, we undertook a realist review to illuminate and understand the relationships and impact of the interaction between components involved in the process of deprescribing [19, 20]. The aim of our review was to construct a programme theory to inform the development of a framework that describes and explains the key components of good practice that supports a person-centred approach to stopping medicines in older patients with polypharmacy. Our research question and objectives are described in Table 1.

**Table 1:** Research question and objectives

**Research question:** How, for whom and in what contexts can safe and effective tailoring of clinical decisions related to medication use work to produce desired outcomes?	
**Research objectives:**	
• To construct a programme theory that describes and explains key components of good practice that supports a person-centred approach to stopping medicines	
• To present recommendations to support policy	

## Methods

The review followed the key steps of conducting a realist review outlined by Pawson et al. [19] of clarifying the scope, searching for the evidence, selecting articles extracting and organising data and synthesising the evidence and drawing conclusions. Realist review is theory-driven configurational approach to evidence synthesis that is commonly used to make sense of complex phenomena (in this case, deprescribing), where outcomes are sensitive to context. Our review followed the current consensus methodological and publication standards for realist syntheses developed by the RAMESES project (www.ramesesproject.org) [21].

### Step 1: Clarifying the scope

**Fig. 1:**
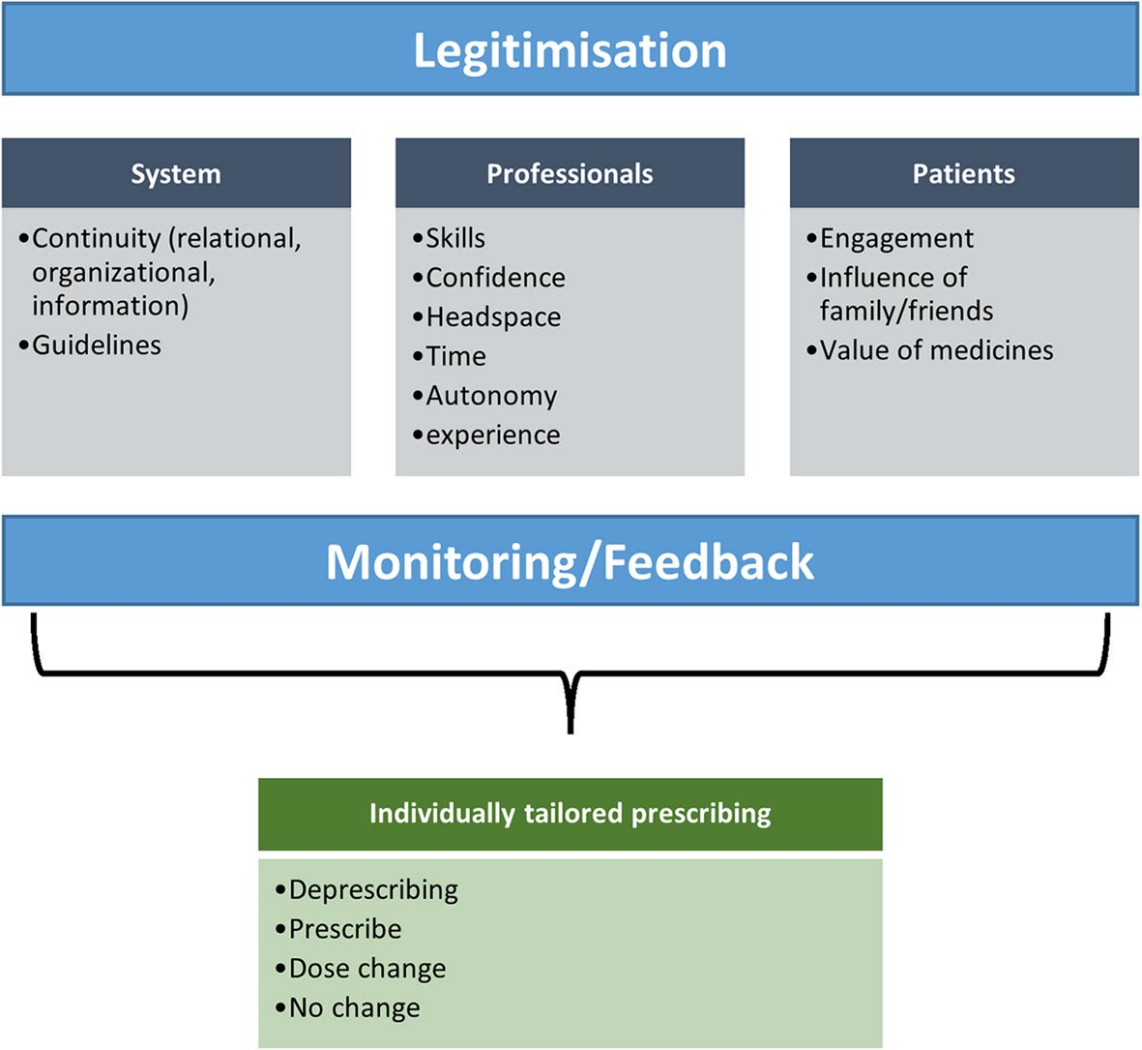
Initial programme theory

Realist reviews begin with the development of an initial “draft” theory of how an intervention is understood to work—a programme theory [21]. Our initial programme theory (Fig. 1) was developed through iterative team discussions and consultation with representatives from key stakeholder groups. The stakeholder group included a mixed audience of clinicians, NHS managers and clinical academics. This group met three times over the duration of the study, during which we presented initial findings and invited stakeholder discussions on the interpretation of findings.

During these meetings, we drew on our content expertise to discuss, debate and identify the key processes and assumptions central to deprescribing. The initial programme theory that we developed set out the concepts and processes we needed to consider in our realist review, as well as the putative relationships between them.

Our initial programme theory informed our review in two key ways. First, our searching strategy was developed to capture the concepts identified with the initial programme theory. Secondly, the concepts identified in the programme theory helped inform our analysis and provided a framework for organising our emerging context-mechanism-outcome-configurations (CMOCs).

As we reviewed the evidence, the initial programme theory was gradually refined by focussing our CMOC development on parts of the programme theory judged as the most important in providing explanations for tailored deprescribing.

### Step 2: Searching for the evidence

Our search strategy was designed to identify relevant literature on the complex intervention that is stopping medication (in the context of individual tailoring of medication use). An information specialist developed a detailed search strategy. We searched the following databases: Ovid MEDLINE, Embase, CINAHL, the Cochrane Library (including the Cochrane Central Register of Controlled Trials (CENTRAL) and Database of Abstracts of Reviews of Effects (DARE)), Cochrane Effective Practice and Organisation of Care (EPOC) Group Specialised Register, Campbell Collaboration Library of Systematic Reviews, JBI Database of Systematic Reviews and Implementation Reports, PsycINFO, Allied and Complementary Medicine Database (AMED) and CAB Abstracts. Details of our searches can be found in the supplementary material. We searched trial registries [22] and grey literature including Google and Google Scholar websites and websites of relevant stakeholders (including RCGP Bright Ideas; National Clinical Guideline Centre; Royal Pharmaceutical Society; conference abstracts, e.g. PRIMM). A detailed search strategy can be found in Additional file 1. We also contacted experts in the field who may be able to signpost us to further literature. We used “pearling”—where we examined the reference list of finally included relevant documents to identify additional ones.

#### Additional searches

This review was part of a larger project that also included a scoping review. We screened the documents included in the scoping review for their eligibility to be included in our realist review. Furthermore, we also screened qualitative studies identified by the scoping review search for their eligibility to be included in the realist review, as we judged these were likely to contain rich information relevant to the development of the programme theory.

### Step 3: Selecting articles and extracting data

Inclusion and exclusion criteria for the review were based on our research question, draft programme theory and team discussions. These are outlined in Table 2.

**Table 2:** Inclusion and exclusion criteria for the realist review

Inclusion criteria	Exclusion criteria
Populations: all participants aged 50 years and over with multimorbidity (two conditions or more)	Studies focused solely on toxicity reactions
Interventions (concepts/process and theory)—any systematic intervention process used to safely withdraw medications in older people with multimorbidity and polypharmacy and the outcomes used to measure the effectiveness of these strategies.	
Context: documents conducted in any appropriate setting (general practice/pharmacy/home setting)	Documents from low- and middle-income countries, studies not published in English
Study design: Any comparative studies including RCTs, cohort or case control studies, qualitative studies and grey literature	

The criteria in Table 2 were only applied to the data set in the first phase of screening conducted at the title and abstract level by AT. A random sample of 10% of these were independently reviewed by KM and GW to help ensure that the criteria were applied consistently, and disagreements were resolved through discussion.

The selection of full-text documents primarily focused on the extent to which the articles could contribute to the development of the programme theory. Documents were assessed on whether they contained relevant data (whether they contributed to programme theory refinement) of sufficient rigour (whether the data contained within the documents were generated by credible and trustworthy methods) [21, 23]. This meant that if a CMOC was based on a limited amount of data, the methods used (if any) within the document were examined in greater detail, and a discussion was had among the review team about whether the evidence presented warranted making changes to aspects of the developing CMOCs and programme theory.

Documents that did not mention patient involvement in the deprescribing/medication management process were judged to be of lower relevance to our research question (because they were less likely to contain data on individually tailored approaches to medication management) and were therefore excluded from the review.

Document characteristics were extracted into an Excel spreadsheet and included full-text documents uploaded into NVivo [24] for data management and analysis by AT.

### Step 4: Synthesising the evidence and drawing conclusions

The coding of relevant extracts from documents was largely inductive, with some deductive coding based on the initial programme theory. The initial stages of coding focused on the conceptual level and classified content into broad descriptive categories. This initial process helped us manage the data as well as make sense of the landscape of the literature and helped us make decisions about whether we had captured enough data to further develop and refine the programme theory.

The second phase of the analysis involved re-examining the broad conceptual level categories and developing context-mechanism-outcome configurations (CMOCs) in order to explain how an outcome was caused by an interaction between the context and a mechanism. A list of potential CMOCs was created by AT and then shared and discussed with GW, JR and KM as well as with our patient and public involvement (PPI) partners. Developing CMOCs were incorporated into the refined programme theory. Diagrams of partial or subsections of the programme theory were created to help guide and illustrate our findings. This process continued iteratively until CMOCs were considered to have sufficient explanatory value (102), namely consilience (when the CMOC was able to account for as much of the possible data related to that CMOC), simplicity (when the CMOC is simple and does not have contain many caveats) and analogy (when the CMOC relates well to what is currently known).

The final stage of analysis involved discussion with key stakeholders and patient and public project partners to identify high-level concepts, which we then incorporated into our final programme theory.

#### Engagement with substantive theory

We drew on formal theories to help substantiate and develop the inferences about the CMOCs we were developing. They also acted as lenses through which to bring together the findings of the review. Some of the theoretical ideas that informed the development of some of the CMOCs were derived from concepts in the documents included in the review (such as trust [25–30], continuity of care [31–35], narrative medicine [36, 37], shared decision-making [38–42] and other theoretical frameworks [43] which were purposively sought to help situate findings within a wider context). These other theories were identified through team discussions, where we drew on the expertise and knowledge of members of the research team and aided the iterative process of programme theory refinement.

## Results

**Fig. 2:**
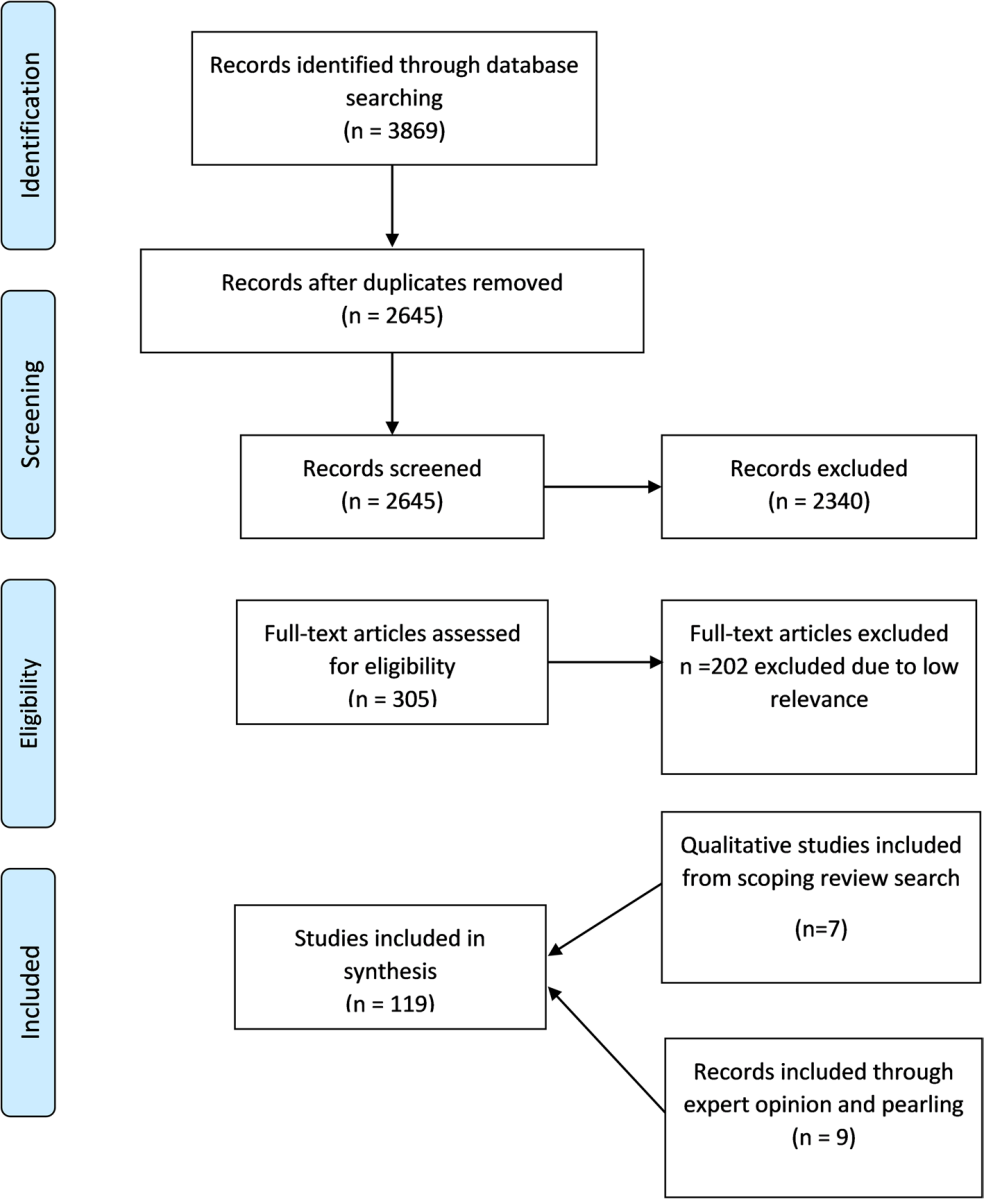
Document selection flowchart

**Fig. 3:**
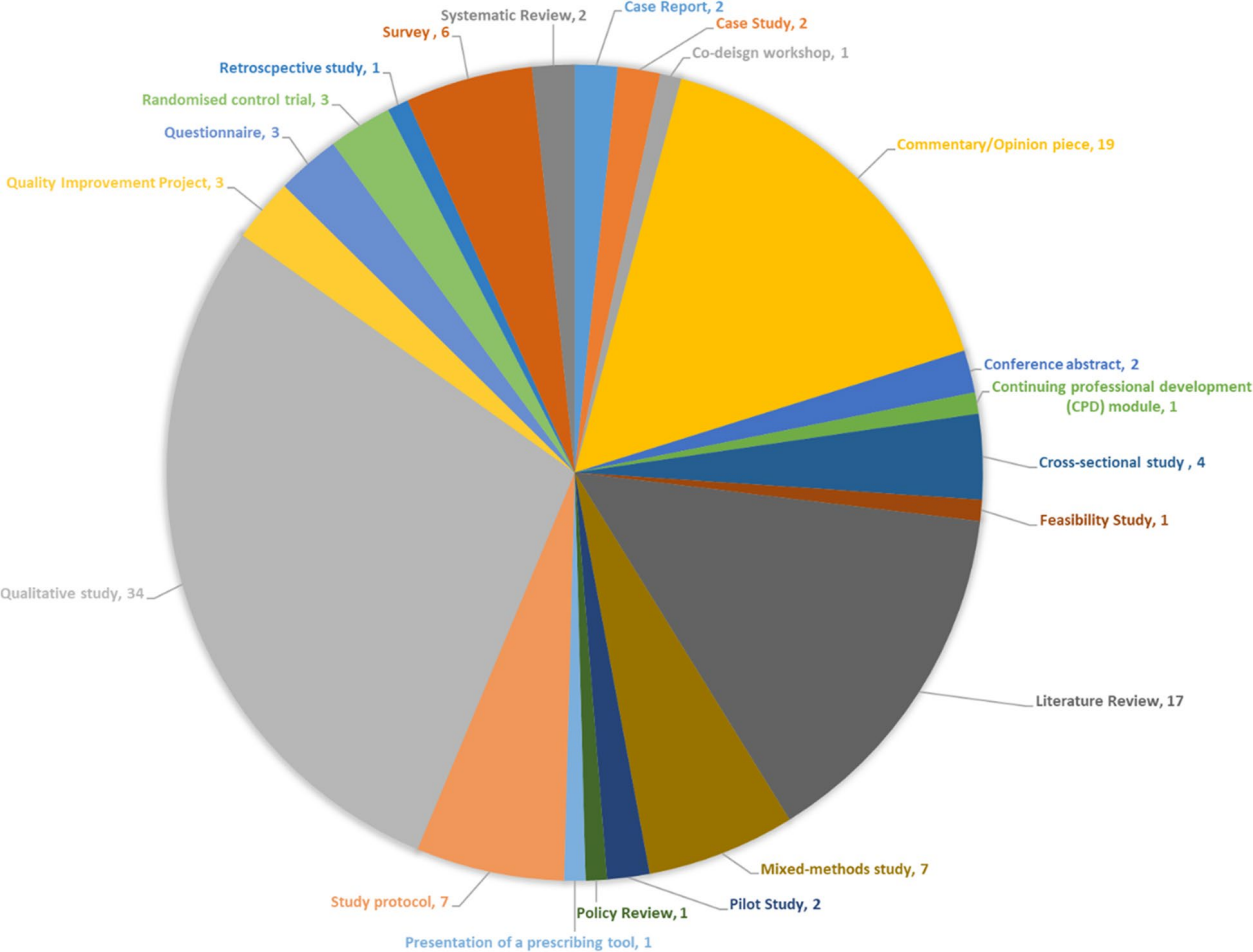
Types of documents included in the review

In total, 119 documents were included in the review (Fig. 2). Documents were published between 1997 and 2020 and included a mixture of study designs and article types (see Fig. 3 and Additional file 2 for detailed table of included studies). The initial search also included documents relating to the topic of medication adherence. We included some of these where we judged they contained relevant data and could help us refine some of our CMOCs about how patients value their medication.

We provide a narrative overview of the different concepts and 34 CMOCs (see Table 3) developed through our analysis (see Additional file 3 for analysis with supporting data). Our analysis can be divided into three sections. The first section describes the deprescribing landscape and explains how different organisational/system, healthcare professional and patient-level factors affect the deprescribing process. The second section builds on the first and identifies and explains potential intervention strategies that may help to navigate the challenges and complexities of tailored deprescribing described in section one. The final section describes our final programme theory that presents four high-level concepts, developed based on sections one and two, to help inform policy and practice.

**Table 3:** CMOCs developed

**Influence of organisational/system-level factors**	
**CMOC1:** In the absence of applicable deprescribing guidelines and evidence **(C)**, healthcare providers may feel like they cannot make justifiable decisions regarding medication changes **(O)** because they don’t feel like these decisions are supported by the system **(M)**.	
**CMOC2:** When healthcare providers feel like they cannot make justifiable decisions that are supported by guidelines **(C)** they may be reluctant to make changes to medications **(O)** because they are afraid of negative consequences **(M)**.	
**CMOC3:** When healthcare practitioners are not supported by incentive and policy structures **(C)** they may not be able to take the time necessary for complex medication management processes **(O)** and be reluctant to make changes **(O)** because they don’t feel supported to do so **(M)**.	
**CMOC4:** When healthcare professionals cannot access information about a patient’s medication regimen **(C)** they do not have an accurate understanding of the medication regimen **(O)** because they don’t understand the patient’s history **(M)**.	
**CMOC5:** When healthcare professionals are unsure about whose responsibility medication management is **(C)** they may struggle to engage in making medication changes **(O)** because they don’t feel they have ownership over the process **(M)**.	
**Influence of healthcare professional-level factors**	
**CMOC6:** When a healthcare professional has previous experience deprescribing medication **(C)** they are more likely to feel able to deprescribe **(O)** because they know what to do and expect **(M)**.	
**CMOC7:** When healthcare professionals feel they don’t have the necessary skills and knowledge to manage medicines in older adults **(C)** they are less likely to make changes to patients’ medicine regimes **(O)** because they are not confident in their ability to make good decisions **(M)**.	
**CMOC8:** When medicines have been prescribed by a specialist **(C)** other healthcare providers from other specialities may be reluctant to make changes to patients’ medicine regimens **(O)** because they do not feel they have the knowledge to make a safe decision **(M**).	
**CMOC9:** When medicines have been prescribed by another healthcare professional **(C)**, healthcare providers may be reluctant to make changes to patients’ medicines **(O)** because they are worried about damaging relationships with the original prescriber as well as between the original prescriber and the patient **(M)**.	
**CMOC10:** When healthcare professionals don’t have dedicated time **(C)** they may be less likely to make changes to patients’ medications **(O)** because they do not have the emotional and cognitive capacity to consider complex issues **(M)**.	
**CMOC11:** When healthcare professionals do not have time **(C)** they may find it difficult to fully consider a patient’s care goals **(O)** because they are forced to prioritise what they spend their time on **(M)**.	
**Influence of patient-level factors**	
**CMOC12**: When patients believe medicines are a sign of good care **(C)** doctors may be reluctant to consider deprescribing **(O)** because explaining and justifying any deprescribing is more emotionally and cognitively taxing (**M**).	
**CMOC13:** When patients believe their medicines are providing them with benefits **(C)** doctors may find it difficult to discuss deprescribing (**O)** because explaining and justifying any deprescribing is more emotionally taxing **(M)**.	
**CMOC14:** When patients believe a medicine might be working or will work in the future **(C)** they are likely to want to continue taking it **(O)** because they hope they are doing something to help their condition **(M)**.	
**CMOC15:** When patients believe their medicines as keeping them alive **(C)** healthcare professionals may find it difficult to discuss deprescribing **(O)** because they don’t want their patients to feel they have abandoned them **(M)**.	
**CMOC16:** When patients view medicines as prolonging their lives **(C)** they may be reluctant to stop taking them **(O)** because they view deprescribing as a sign that they aren’t worth keeping alive anymore (**M)**.	
**CMOC17**: When patients believe medicines are providing them with benefits **(C)** patients may be reluctant to discontinue them **(O)** because they are afraid of negative consequences **(M)**.	
**CMOC18:** When families or carers perceive medicines to have a benefit for the patient **(C)** healthcare professionals may be reluctant to consider deprescribing **(O)** because they feel pressured not to do so **(M)**.	
**CMOC19:** When families/carers are involved in a patient’s healthcare **(C)** patients may be more able to engage in decision-making about their medicines **(O)** because they feel supported by them **(M)**.	
**Shared decision-making**	
**CMOC20:** When healthcare professionals involve patients in the medication management process **(C)** they are more likely to make better decisions about medication **(O)**, because of their shared expertise **(M)**.	
**CMOC21:** When healthcare professionals are aware of a patient’s perspectives and beliefs about medicines and their goals of care **(C)** they are more likely to achieve patient-centred outcomes **(O)** because the patient is understood **(M)**.	
**CMOC22:** When healthcare professionals involve patients in the decision-making process **(C)** they are more likely to make defendable decisions about medications **(O)**, because of their shared responsibility **(M)**.	
**Continuity of care and development of trust**	
**CMOC23:** When patients are presented with conflicting recommendations about their medication by health care professionals **(C)**, their trust may decrease **(O)**, because they don’t know who to believe **(M)**.	
**CMOC24**: When patients and their carer/family are asked to change their usual medication by a health care professional they are unfamiliar with **(C)**, they may be reluctant **(O)**, because they are concerned the person does not know what is best for them personally **(M)**.	
**CMOC25:** When a health care professional demonstrates to a patient they understands their needs and goals **(C)** the patient is more likely to trust them **(O)** because they believe the heath care professional is acting in their best interest **(M)**.	
**CMOC26:** When a patient trusts their healthcare provider **(C)** they may be more likely to consider changes to their medication **(O)** because they believe their healthcare professional is acting in their best interest **(M)**.	
**CMOC27:** When healthcare professionals know that they will be able to follow-up a patient **(C)**, they are more likely to try deprescribing **(O)**, because they are reassured they will be able to manage potential harms **(M)**.	
**Monitoring**	
**CMOC28:** When a clinician judges that a patient may benefit from a change in medication **(C)**, they are likely make small incremental changes **(O)** because they are concerned about causing harm to the patient **(M)**.	
**CMOC29:** When a harms minimisation process is provided by clinicians during medication changes **(C)**, patients are more willing to make these changes **(O)**, because they feel reassured **(M)**.	
**CMOC30:** When a patient provides feedback to a clinician about the effects of a medication change **(C)**, they are more likely to make an informed decision about its value **(O)**, because of their new knowledge **(M)**.	
**CMOC31:** When healthcare professionals are aware of a patient’s current perspective and beliefs about their medication **(C)**, patients are more likely to consider medication change **(O)** because they feel understood **(M)**.	
**Multidisciplinary approach**	
**CMOC 32:** When healthcare professionals can draw on the skills and expertise of colleagues **(C)** they feel more confident in making prescription changes **(O)** because they feel re-assured that they are making safe and optimal prescribing decisions **(M)**.	
**CMOC33:** When healthcare professionals can discuss complex cases with colleagues **(C)** they feel more confident about making medication changes **(O)** because they feel supported **(M)**.	
**CMOC34:** When healthcare professionals work collaboratively **(C)** they can improve continuity of care **(O)** and their understanding of their patients’ needs **(O)** because they can share workload **(M)**.	

### Section one: The deprescribing landscape (CMOCs 1–19)

The factors that shape deprescribing can broadly be grouped into organisational/system-level factors, health care provider-level factors and patient-level factors. These different factors interact with each other both within and across different levels to produce different outcomes and affect the ways in which healthcare practitioners and patients are able to engage with the deprescribing process.

### Organisational/system-level factors

Our review identified three key areas at the organisational/system level which impact the ways in which healthcare providers and patients engage with deprescribing. These include guidelines and policies, transitions in care and difficulty accessing patient information and unclear roles and responsibilities.

While numerous guidelines for medication management exist, these can be difficult to apply and may limit healthcare professionals’ willingness or ability to consider deprescribing. For example, medication guidelines are often based on the management of single conditions and on evidence from trials in younger populations (103), thus making them difficult to apply in older patients with multimorbidity. This can limit the extent to which healthcare practitioners feel comfortable tailoring medicines to individual patients and make it difficult to feel that they are making safe and/or defendable decisions (CMOCs 1 and 2). This is compounded by incentive and administrative structures that can limit the time available to practitioners to be able to undertake the complex process of deprescribing (CMOC3, 10 and 11).

The management of multimorbidity often involves multiple prescribers and transitions between primary and secondary care. These transitions can result in poor documentation of changes made to patient treatment plans and, as a consequence, can limit healthcare professionals’ understanding of patients’ current medication regimens and health needs [44], therefore hindering the process of medicines optimisation and deprescribing (CMOC4).

The literature reveals disparities in opinion among healthcare professionals regarding who the assignment of medication management responsibilities should fall to [45]—whether it should fall within the remit of specialists or general practitioners. This lack of a clear “clear line of responsibility” [26] may leave healthcare professionals feeling like they do not have ownership of the process, therefore making them reluctant to engage with it (CMOC 5).

### Healthcare provider-level factors

A number of individual and interpersonal factors such as skills and level of experience, professional etiquette and time and “headspace” (cognitive and emotional capacity) can shape the ways in which healthcare professionals are able to undertake the complex decision-making involved in deprescribing.

Medication management in older people experiencing multimorbidity is seen to require specialised skills and knowledge of the physiological changes associated with ageing [46]. When healthcare professionals feel they lack the skills in what they see as an area that requires specific training or experience, they may not feel confident making changes to patients’ medicines (CMOC7), particularly if these have been prescribed by a specialist (CMOC8). More experienced healthcare professionals may feel more comfortable making recommendations because they have had to balance quality of life against risks and benefits of medicines before and know what to do and expect (CMOC6).

The involvement of multiple healthcare professionals working across different specialities and healthcare settings can present a number of interpersonal challenges for deprescribing. Healthcare professionals may be reluctant to deprescribe medicines prescribed by another prescriber out of fear of damaging working relationships (CMOC9). They may also be worried about damaging the relationship between the patient and the original prescriber by presenting patients with conflicting recommendations and potentially damaging patient trust (CMOC23).

Healthcare professionals operate within contexts that limit the time they are able to dedicate to the complex decision-making process necessary for safe deprescribing. This can affect healthcare professionals’ headspace (cognitive and emotional capacity) needed to balance the potential benefits and harms of medication changes and to be able to engage with patients to deliver tailored person-centred care.

### Patient-level factors

A number of documents in our review suggest that patients are open-minded about deprescribing and may be willing to discontinue one or more medications considered “inappropriate” or unnecessary [26, 44, 47–49]. However, patients’ willingness to engage with and consider deprescribing may be shaped by how they perceive the value of their medications and the involvement of their families and carers.

Medications can carry a symbolic value for patients, and this has a number of implications for the deprescribing process. Medicines can be perceived as a symbol of good care and healing [50, 51]; a means of maintaining identities, independence and daily life; and a symbol of hope where medication may improve the patients’ health in the future. In the context of these beliefs, patients may perceive deprescribing as a withdrawal of care or abandonment, making the justification of deprescribing cognitively and emotionally taxing for healthcare professionals (CMOC12, 13 and 15).

Patients’ families and carers can also influence the medication management process. Their expectations may make it more challenging for healthcare professionals to have conversations about realistic goals of care and can put pressure on healthcare professionals to maintain patients’ medication regimens (CMOC18). However, families and carers can also be an essential source of support for patients by helping them access information and supporting them to be more actively engaged in their care and decisions about their medicines (CMOC19).

### Section two: Potential intervention strategies to support tailored deprescribing

**Fig. 4:**
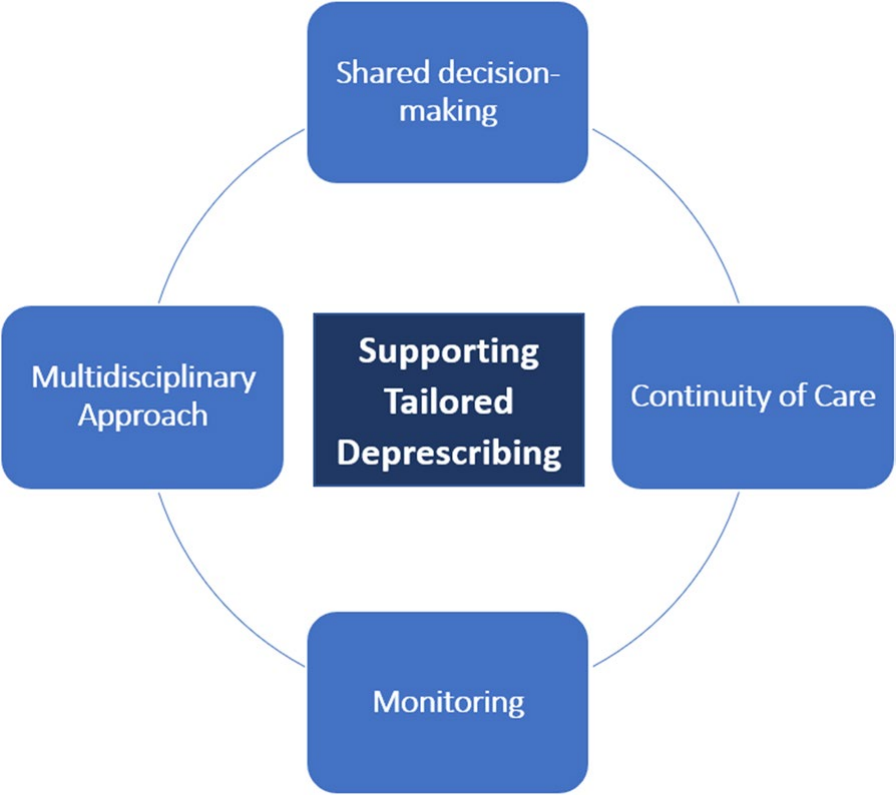
Potential intervention strategies to support tailored deprescribing

Section one described the interrelated and complex system of factors within which deprescribing and medication management, in general, take place. Our analysis identified potential intervention strategies and contexts that may need to be present to mitigate some of the challenges described above. These intervention strategies include shared-decision making, continuity of care and the development of trust, monitoring and a multidisciplinary approach (see Fig. 4). They work by modifying some of the contexts presented in CMOCs 1–19 to trigger the mechanisms necessary to produce desired outcomes.

#### Shared decision-making

Shared understanding and decision-making, where both healthcare professionals and patients actively participate, share information and reach a consensus on what is wrong and what can be done, was identified as an important strategy in the management of problematic polypharmacy and deprescribing in a number of documents included in our review. It enables collaborative decision-making between patients and healthcare professionals and recognises patient experiences and expertise, helping them to jointly navigate some of the complexities and uncertainties of deprescribing in the context of wider management of multimorbidity and everyday life.

Shared-decision making can help establish treatment priorities and ensure changes are sensitive and responsive to the context of patients’ lives and medication beliefs (CMOCs 12–19). Medications can have a number of symbolic meanings attached to them that can make it difficult for both patients and healthcare professionals to consider the option of deprescribing (CMOCs 12–18). By becoming aware of these beliefs and goals of care by involving patients in the medication decision-making and management process, healthcare professionals may be more likely to achieve patient-centred outcomes (CMOC21). Collaborative sense-making and decision-making with patients can also allow healthcare professionals to share the responsibility for deprescribing and help them make defendable decisions (CMOC22), thus addressing some of the challenges of navigating guidelines laid out in CMOCs 1 and 2.

#### Continuity of care and development of trust

Continuity of care was a prominent theme identified across the literature as an important feature of tailored medication management. Continuity of care has been defined as “The extent to which a person experiences an ongoing relationship with a clinical team or member of a clinical team and the coordinated clinical care that progresses smoothly as the patient moves between different parts of the health service” [52]. The literature distinguishes between three main types of continuity of care—informational, management and relational continuity. Informational continuity refers to the use of information on past events and personal circumstances to make patient-centred decisions; management continuity involves a consistent and coherent approach to the management of a patient’s changing needs; and relational continuity refers to the ongoing relationship between a patient and one or more health providers. Each of these types of continuity can be important for the building and maintenance of trust between patients and healthcare professionals by providing the opportunity to amass experiences of trustworthy behaviour and establish norms of cooperation and reciprocity [53], which can ultimately contribute to the effectiveness of medical care [53].

Within the context of medication management, lack of continuity resulting from siloed care and difficulties accessing patient information can influence whether healthcare professionals make changes to patients’ medications (CMOC4) and can damage patient trust (CMOC 23). Improved continuity of care can help reassure patients that they are being managed by a professional who understands their personal situation (CMOC26), making them more likely to consider medication changes recommended by that professional (CMOCs24 and 25). Management continuity can also help reassure patients and healthcare professionals that any unwanted effects of medications changes will be managed, therefore making them more likely to consider making changes such as deprescribing (CMOC27).

Continuity of care plays an important role in building trust between patients and healthcare professionals (CMOCs 23–27), which can make the process of deprescribing less emotionally taxing for healthcare professionals (CMOCs 4, 12, 13), and make patients more likely to their recommendations when their beliefs are challenged (CMOCs 14–18). The trust engendered through continuity of care may also help facilitate shared-decision making described above (CMOCs 20–22).

#### Monitoring and learning

Deprescribing entails a number of complexities and inherent uncertainties for both healthcare professionals and patients (CMOCs 1–19). A clear follow-up or monitoring process following changes to patients’ medication regimens may help alleviate some of the concerns about potential negative consequences and withdrawal of care (CMOCs 15–17). A monitoring process may work to reassure patients and healthcare professionals that both parties will work together to learn from changes made and address any potential negative effects (CMOC28 and 29 and related to CMOC27); provide an opportunity to consider changing patient perspectives and priorities (CMOC 31); and provide an opportunity for patient feedback to inform the deprescribing process (CMOC30). Furthermore, monitoring may also contribute to continuity of care and thus further enhance the development and use of trust (CMOC 27).

#### Multidisciplinary approach

Working in multidisciplinary teams may aid the process of deprescribing in a number of ways. Being able to draw on the expertise and experience of colleagues (CMOCs 31 and 32) may help overcome some of the challenges healthcare professionals face when they lack confidence in their own skills and experience (CMOCs 5–7). Discussing complex cases with colleagues may help healthcare professionals feel supported and reassured that planned medication changes are safe and defendable (CMOCs 31 and 32), including in the absence of adequate guidelines (CMOC1). Collaborative working allows healthcare professionals to share the responsibility and workload of deprescribing (CMOC 34), therefore potentially mediating some of the challenges of limited time (CMOCs 10 and 11).

Finally, multidisciplinary team working may help contribute to informational continuity of care (CMOC 33) and thereby help to improve patient trust (CMOC 25).

### Section three: A final framework to inform policy and practice—the DExTruS framework

**Fig. 5:**
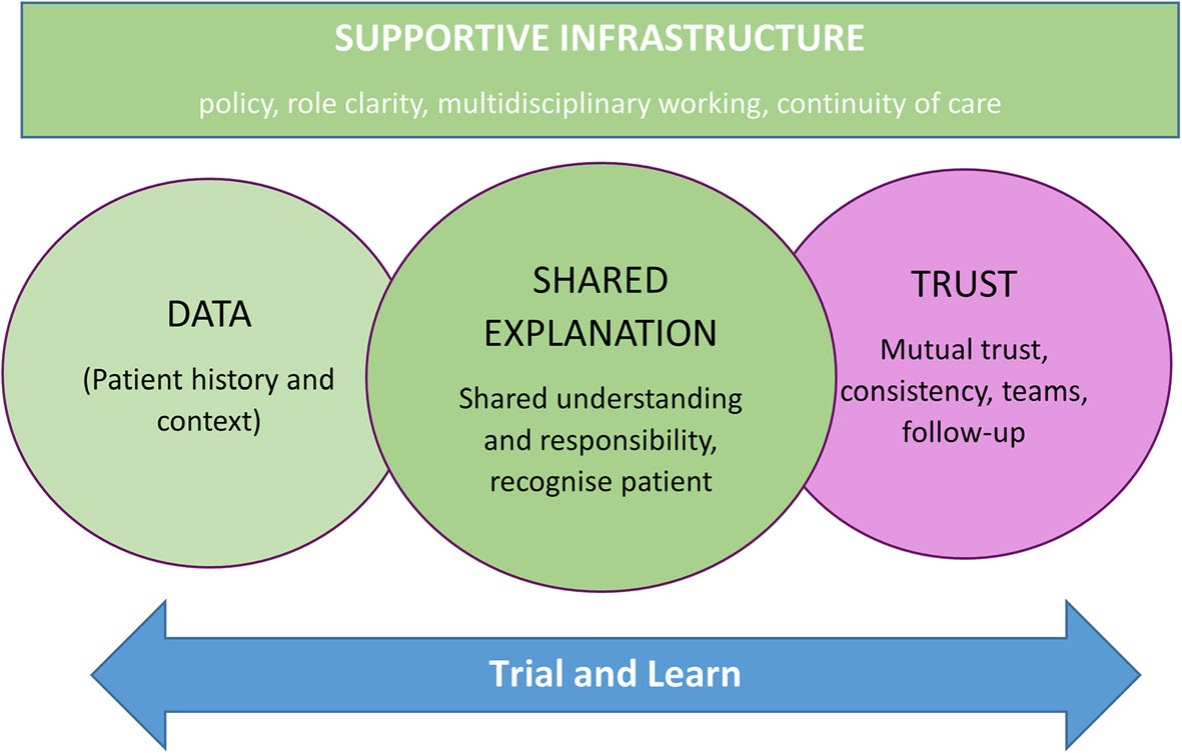
Final programme theory—the DExTruS framework—core elements needed to support effective tailored prescribing through addressing barriers of cognitive and emotional load

Based on the findings discussed above, further discussions with stakeholders and PPI contributors helped identify four high-level concepts that fit into a final programme theory. Our final programme theory recognises that problematic polypharmacy requires what we have called a tailored approach to medication management or deprescribing. Our analysis in previous sections highlights that tailored prescribing often involves beyond-protocol decision-making and complex emotional and cognitive work for clinicians and patients. Our final programme theory describes four key concepts—*supportive infrastructure* that provides opportunities for trial and learning; consistent access to high-quality, relevant *data* (patient history and context); creating a shared *explanation* of the meaning and purpose of medications; and building *trust* (Fig. 5 and Table 4)—needed to enable tailored prescribing through addressing the cognitive and emotional load of deprescribing. This can be summarised as the DExTruS framework (*D*ata, *Ex*planation and *T*rust) occurring within a *S*upportive infrastructure that supports opportunities for trial and learning.

**Table 4:** Detailed programme theory—this table provides a detailed explanation of the DExTruS framework shown in Fig. 5 (numbers in brackets refer to CMOCs)

What to do	Why do it?	Anticipated outcomes
**SUPPORTIVE INFRASTRUCTURE** ▪ Policy and incentive structures (1, 2) ▪ Clarity of professional roles (5) ▪ Building skills and confidence in primary care clinicians (6–8) ▪ Recognising distinct generalist and specialist expertise equally and enable ways to work in MULTIDISCIPLIANARY TEAMS (32–34) ▪ CONTINUITY OF CARE (23–27, 29)	Provides PERMISSION and so motivation of and prioritisation for staff (3, 10, 11) Reduces concerns from making changes (2) and cognitive and emotional load (1–8) Increases knowledge needed to make decisions (6–8) Allows healthcare professionals to draw on a broader range of expertise (32) and share workload (34) Overcomes professional inertia associated with uncertainty (7, 8) and concern about professional relationships (9) Healthcare professionals feel more confident and supported (32, 33, 34) and able to manage potential harms (27) Enhances patient **TRUST** (23–25) and may help facilitate shared-decision making (20–22)	Enhanced **TRUST** between patients and healthcare professionals Patients more likely to consider changes Reduced medication related anxiety/fear Achieve patient-centred outcomes Patient and professional satisfaction
**CONSISTENT ACCESS TO HIGH QUALITY RELEVANT DATA** ▪ Contextual data: what meds, why, in context of individual patient (4) ▪ Informational CONTINUITY OF CARE (23)	Enhances **TRUST** between patient, their carers and healthcare professionals (23–25)	
**SHARED EXPLANATION of meaning and purpose of medications in the context of daily living, recognising and acting on patients’ lived experiences and priorities** ▪ Recognise and (re)frame meaning and value of meds with patients (12, 14, 15) ▪ SHARED DECISION MAKING (20–22): Recognise/negotiate expertise of patient and family (19) to support sharing the load (20), understanding (21) and responsibility (22).	Recognises patient’s agendas and their implications (12–19) Avoids patient perceptions of abandonment (15, 16), maintains hope, optimism (14) Builds patient/family **TRUST** through a shared sense of working for “my best interests” (23–26) Shared understanding (21) and responsibility (22) with patient and family, which may help to make defendable decisions (1 and 2).	
**TRIAL AND LEARN** ▪ MONITORING (28–31)—tailored prescribing through incremental change (28), harm minimisation (29), with follow-up and CONTINUITY (27, 30,31)	Enables patient perspectives to be heard following changes (30,31) and may enhance **TRUST** (31) Overcomes professional inertia associated with uncertainty of outcomes (7) and fear of negative consequences (15–17)	

#### Supportive infrastructure

The management of complex multimorbidity and polypharmacy is an inherently uncertain and complex process. If healthcare professionals are to manage the uncertainties of deprescribing, they require guidance to be supportive of this complex work. Current policy recognises a principle of person-centred care as the gold standard and tailored care as good practice [1, 3, 5, 54]. While existing guidance describes the steps within the consultation that support good practice around deprescribing [55], it does not address the organisational context and elements needed to avoid the negative impact on professionals’ confidence and ability to deprescribe described within our work (CMOCs 1–5). A supportive infrastructure and guidance, therefore, also need to offer a framework outlining “safe boundaries” for uncertain practice and the resources needed to deliver this complex role. These may include clarity around whose remit tailored de(prescribing) falls under (CMOC 5); recommendations on time and resource allocation to undertake this complex task (CMOCs 10 and 11).

A supportive infrastructure may also be important for legitimising the deprescribing role. Professionals taking on deprescribing may be described as having “boundary spanning” roles by needing to negotiate and reconcile different sources of information and perspectives (CMOCs 12-17) as well as develop and coordinate a plan to manage these changes (CMOCs 28–31). Boundary spanners relate practices in one field to practices in another by negotiating the meaning and terms of the relationship between them [56]. In order for boundary-spanning roles to be effective, they need to be seen as legitimate within the system around them [57]. By clarifying whose responsibility medication tailoring and (de)prescribing is and allocating sufficient resources to the role, a supportive infrastructure may act to formalise and legitimise the deprescribing role, thereby granting healthcare professionals the “permission” to undertake it.

Another key form of support for healthcare professionals undertaking tailored medication management and (de)prescribing may come from a collaborative and multidisciplinary approach. Our analysis has highlighted that being able to work with colleagues to manage deprescribing may allow them to draw on the expertise of their colleagues (CMOCs 31 and 32), as well as share workload and responsibility (CMOC 34).

#### Consistent access to high quality and relevant data

While health systems routinely collect large amounts of data, our review highlighted that consistent and easy access to the right kind of data matters in the context of tailored (de)prescribing (CMOC4). Healthcare professionals require access to contextual data—patient history and context (CMOCs 15–17), what the anticipated impact of medications are, how are these impacts judged and what conversations have been had with the patient—to be able to confidently undertake the complex and uncertain process of tailored medication decision-making (CMOC4). The availability of this data may help support shared-decision making (CMOCs 20–22) and may promote informational continuity (CMOC 23 and 25), which can be vital in supporting patient trust (CMOC 23 and 25).

#### Shared explanation and understanding of values/beliefs and purpose of medicines

Tailored prescribing decisions rely on understanding patients’ experiences, values and beliefs about the role of their medicines within their wider context of their healthcare and daily living (CMOCs12–18). An enhanced process of shared-decision making may help facilitate this shared understanding and explanation between healthcare professionals and patients needed to achieve tailoring (CMOCs 20–22) and tailored explanations. These explanations can convey to the patient that the professional understands them (CMOC 24) and is acting in their best interests (CMOC 26).

In creating a shared understanding, it is necessary to ensure that patients do not receive conflicting information from different healthcare professionals to avoid undermining patient trust (CMOC 23). As discussed above, this may require access to and coordination of high-quality contextual data and a multidisciplinary and collaborative approach to medicines management.

#### Trial and learn

#### Trust

Trust, a feeling of confidence or reassurance that the healthcare professional has the patients’ best interests at heart and that their decisions are grounded in an understanding of the patient, is a core component of effective healthcare. It can be particularly central when managing uncertainty [58–60] which remains one of the key challenges in managing multimorbidity and polypharmacy. It runs as a golden thread through the final programme theory. Our analysis identified key elements involved in developing trust in the context of tailored (de)prescribing. Professional trust in their own decisions (confidence), which is backed by a supportive infrastructure and shared responsibility for decision-making (CMOCs 1–5, 7 and 8). Patient trust in the healthcare professional can be supported through producing a shared understanding of medicines and tailored explanations (CMOCs 12–18 and 23–27), through a consistent management approach which minimises conflicting advice and includes a planned follow-up to review changes (CMOC 27) based on patient feedback (CMOC 30).

### Alignment with formal theories and other literature

To help situate our findings within a broader context and to help support any inferences made when constructing our final programme theory, we drew on formal or substantive theories and other literature. These include *conservation of resource theory and loss aversion bias*, *social support theory* and *therapeutic relationships.*

#### Conservation of resource theory and primacy of resource loss bias

Conservation of Resource Theory postulates that individuals are motivated to protect current resources and acquire new ones [61]. Resources can be loosely defined as anything that can help an individual attain their goals, such as objects, relationships and social support [61]. The value people attach to different resources varies based on their experiences and the contexts of their lives [61]. Following from the principle of conservation and acquisition is the primacy of resource loss which theorises that it is more psychologically challenging for individuals to lose resources than it is beneficial for them to gain the resources they have lost [61].

Deprescribing involves a deviation from the status quo and removing or losing medications (resources) that patients perceive as bringing them value (CMOCs 12–18). Conservation of resource theory and the primacy of resource loss bias may help to explain the inertia experienced by healthcare professionals and patients when considering deprescribing.

#### Social support theory

Social support refers to the broad range of processes through which social relationships promote health and wellbeing [62]. Information that leads individuals to believe that they are cared for and belong to a network of communication is also a form of social and can contribute to resilience in times of stress [63].

The complexity and uncertainty involved in deprescribing can be psychologically and emotionally taxing for both healthcare professionals and patients (CMOCs 12, 13, 16, 17). Our review identified a number of mechanisms related to professionals’ and patients’ need to feel supported and understood in order for desired outcomes to be achieved (CMOCs 1, 2, 19, 21, 31 33, 34).

Our final programme theory describes the importance of a supportive and enabling infrastructure which may provide healthcare professionals with the support and resources necessary to manage the uncertainties and complexities of deprescribing. Similarly, consistent access to data, creating shared understandings and building trust may support patients by reassuring them that healthcare professionals understand and value their needs and priorities.

#### Therapeutic relationships

Positive relationships between patients and healthcare professionals can improve patient satisfaction, professional fulfilment, compliance with prescribed treatments, saving time and reducing the number of patient complaints [64].

Our review highlights that the relationship between healthcare professionals and patients may be central in supporting tailored deprescribing (CMOCs 25 and 26). Positive relationships, where trust is generated and maintained, may be key to navigating the uncertainties [32] entailed by the deprescribing process. However, the centrality of this relationship may also act as a barrier to achieving medication changes. Healthcare professionals may be worried about damaging the therapeutic relationships with the patient [65] (CMOC9) and may find it challenging to propose changes when they know these do not necessarily align with patient medication beliefs (CMOCs 12–18). Shared decision-making may be key in negotiating some of these potential tensions in order to reach desired outcomes (CMOCs 20–22).

Trust has been described as one of the most important components of well-functioning relationships [66] and may also contribute to better healthcare outcomes [67]. Continuity of care is associated with higher levels of trust between patients and healthcare professionals. Our analysis has emphasised the importance of all forms of continuity (relational, informational and management) for the building of trust necessary for successful engagement in deprescribing (CMOCs 23–26).

## Discussion

Our review recognises the significant cognitive and emotional load involved for patients and professionals involved in the process of tailored deprescribing and medication management. The necessitated complexity of working “beyond guidelines”, managing uncertainty and maintaining a continuity of approach and trust, if inadequately recognised and supported, can contribute to inertia, resulting in both the patient and healthcare professionals maintaining a prescribing status quo. If we are to achieve the goals set up in the Kings Fund report [1], and most recently in the recent report on overprescribing by the UK Department of Health and Social Care [68], we need to revise our understanding of what good prescribing practice looks like, and so redesign the practice context in which it is delivered.

Our final programme describes key components needed to support this redesign in order to manage or reduce this load and so support tailored deprescribing—summarised in our DExTruS approach. These key components include a supportive infrastructure that provides clear guidance and clarity around professional responsibilities and that enables multidisciplinary working and continuity of care; consistent access to high quality relevant contextual data; the need to support the creation of a shared understanding of the meaning and purpose of medicines; and a trial and learn approach that provides space for monitoring and continuity. Together these components may support the development of trust, which may be crucial to managing and dealing with the inherent uncertainty involved in tailored deprescribing and, in turn, help to optimise outcomes.

The National Institute for Health and Care Excellence (NICE) Medicines Optimisation Guidance outlines the principles that should be followed to optimise medication management [69]. This includes the use of structured medication reviews that incorporate patient views and preferences, decision aids to involve patients in decision-making, and clinical decision-making support. Our previous research identified several practical barriers to delivering those approaches [4]. These include lack of time, energy and headspace among healthcare professionals, and concerns around making and recording defendable decisions.

A review of available deprescribing tools [70] concluded that while existing tools may help address some of the barriers of deprescribing, these do not take into account the multifaceted nature of these barriers which, when considered individually, may not be easy to overcome. Our review highlights the complexity and multifactorial nature of these barriers. It provides insight and explanation into the changes that may be required to address them, described in the elements of the DExTruS framework.

### Strengths and limitations

We conducted our review systematically in accordance with the RAMESES quality standards [21]. The programme theory developed in iterative stages through in-depth, reflective discussions within the project team as well as with patient and public partners and stakeholder groups. The project team and stakeholder group included individuals with varied academic and clinical backgrounds; this multidisciplinary input played an important role in confirming and refining aspects of the programme theory.

Reviews rely on the evidence that is available. The documents included in our review discussed the many barriers and facilitators, and attitudes toward medication management and provided a good overview of the factors influencing the engagement with deprescribing in a broad sense. However, we found that individual deprescribing interventions were often not described in enough detail to be able to draw conclusions on how their different components resulted in desired outcomes. This limited our ability to analyse the role and effectiveness of specific intervention components in producing desired outcomes. However, through analysing the 119 documents included in our review, we have been able to explain the interrelationships between the factors that shape engagement with tailored deprescribing, and we have been able to identify the contexts that may need changing in order for tailored deprescribing to be optimised.

### Implications for practice and policy

Our findings recognise the complex interpretive practice and decision-making involved in decisions with patients and carers about medication use and identify key components needed to support best practice in tailored medication management.

Tailored decision-making requires consistent access to an appropriate range of data, including the contextual data that supports the interpretation of medication's meaning, purpose and value beyond the account offered in a condition-specific guideline. Data includes the patient/carer experience and expectations gathered during the consultation. It also entails an understanding of context, including patient circumstances and their access to other forms of support/services that may offer an alternative to medicines used in health management [68]. Furthermore, this may include the need for an appreciation of the broader non-biomedical literature on medicine use that is not included within condition-specific guidelines [71].

These findings have implications for how we design “medication review” consultations (to allow for patient history and contextual data generation and collection); for professional training (to support the different skills set needed to undertake tailored medication reviews); and for patient records/data management (currently focused in general practice on Quality and Outcomes Framework/disease monitoring and contractual-systems needed). Further research is required to consider how these findings can be implemented and formally evaluated in practice at scale.

Generating shared understanding and meaning of the purpose of medicines in the context of individuals’ daily living involves a two-way process of explanation and negotiation. Tailored decisions are not about telling patients what to take and how but about co-constructing shared explanations of health and illness and negotiating the role and use of medication with that in mind. This work, including our PPI discussions, highlights that these conversations need to start at the beginning of medication use—when negotiating starting medicines with patients—not just when considering stopping.

Trust, defined as a feeling of confidence or reassurance that the healthcare professional explanations and decisions are grounded in an understanding of the patient, is recognised by our analysis as both a key element of tailored prescribing and an outcome potentially developed/supported by good practice in this area. Dealing with the cognitive and emotional impact of deprescribing for both professionals and patients requires shared practice and trust between all parties and opportunities for trial and learn. There are implications for how we support patients, professionals and teams in developing the skills and confidence in constructing trusted explanations [72].

Furthermore, there are implications for how we build “integrated care teams”, share data and decisions between teams, and over time. We suggest that building and maintaining trust is a core component in understanding best practice—shaping how we train professionals and monitor practice. General practitioners have traditionally prioritised the maintenance of the doctor-patient relationship, but maybe we need to refine this to focus on the task of trust. The control of decision-making needs to be with professionals, with the system around them operating as a guide and support—a supportive infrastructure.

### Recommendations for future research

Our work demonstrates and supports the need to expand research on the management of conditions to include a focus on whole person prescribing and the related care processes that are embedded within a wider healthcare context. There is clearly still a need for experimental studies to describe the absolute and relative benefit of given medications in different conditions—providing one important (if insufficient) data source for the negotiations we have described. However, if we want to understand “real-life” prescribing *practice*—the activities and actions of both patients and professionals we need to use complex intervention approaches with explicit attention to the theoretical underpinnings of the intervention. Future research could look to explore how the elements of the DExTruS may be best implemented into practice.

## Conclusions

Our realist synthesis has enabled us to develop a novel framework outlining key components of good practice to support a person-centred approach to stopping medicines in older people with polypharmacy. It highlights that the management of problematic polypharmacy through deprescribing is a complex intervention influenced by system, healthcare professional and patient-level factors. Addressing these factors requires a new approach to deprescribing, which we have encapsulated in the novel DExTruS (Data, Explanation, Trust and Support) framework. The DExTruS framework gives healthcare professionals a framework to analyse their current practice and consider possible gaps in how they deliver tailored prescribing and therefore identify what issues can be addressed locally.

## Supplementary Information

**Additional file 1.** Search strategy.

**Additional file 2.** Table of included documents.

**Additional file 3.** CMOCs with supporting data extracts.

## Data Availability

Datasets used in the review are available from the corresponding author upon request.

## Abbreviation

CMOCs: Context-mechanism-outcome configurations

## References

[1] Duerden M, Avery T, Payne R. Polypharmacy and medicines optimisation: making it safe and sound. London; 2013. Available from: www.kingsfund.org.uk

[2] Krska J, Katusiime B, Corlett SA (2017). Validation of an instrument to measure patients’ experiences of medicine use: the Living with Medicines Questionnaire. Patient Prefer Adherence.

[3] Reeve E, Gnjidic D, Long J, Hilmer S (2015). A systematic review of the emerging definition of “deprescribing” with network analysis: implications for future research and clinical practice. Br J Clin Pharmacol.

[4] Reeve J, Britten N, Byng R, Fleming J, Heaton J, Krska J (2018). Identifying enablers and barriers to individually tailored prescribing: a survey of healthcare professionals in the UK. BMC Fam Pract.

[5] Barnett N, Kelly O (2017). Deprescribing: is the law on your side?. Eur J Hosp Pharm Sci Pract.

[6] Pitkala KH, Suominen MH, Bell JS, Strandberg TE (2016). Herbal medications and other dietary supplements. A clinical review for physicians caring for older people. Ann Med.

[7] Townsend A, Hunt K, Wyke S (2003). Managing multiple morbidity in mid-life: a qualitative study of attitudes to drug use. BMJ.

[8] Vandermause R, Neumiller JJ, Gates BJ, David P, Altman M, Healey DJ (2016). Preserving self: medication-taking practices and preferences of older adults with multiple chronic medical conditions. J Nurs Scholarsh.

[9] Knowles S, Hays R, Senra H, Bower P, Locock L, Protheroe J (2018). Empowering people to help speak up about safety in primary care: using codesign to involve patients and professionals in developing new interventions for patients with multimorbidity. Health Expect.

[10] Wallace E, Salisbury C, Guthrie B, Lewis C, Fahey T, Smith SM (2015). Managing patients with multimorbidity in primary care. BMJ.

[11] Fearon D, Hughes S, Brearley SG (2018). A philosophical critique of the UK’s National Institute for Health and Care Excellence guideline “Palliative care for adults: strong opioids for pain relief”. Br J Pain.

[12] Beers MH (1997). Explicit criteria for determining potentially inappropriate medication use by the elderly. An update. Arch Intern Med.

[13] O’Mahony D, O’Sullivan D, Byrne S, O’Connor MN, Ryan C, Gallagher P (2015). STOPP/START criteria for potentially inappropriate prescribing in older people: version 2. Age Ageing.

[14] Denford S, Frost J, Dieppe P, Cooper C, Britten N (2014). Individualisation of drug treatments for patients with long-term conditions: a review of concepts. BMJ Open.

[15] Reeve J, Dowrick CF, Freeman GK, Gunn J, Mair F, May C (2013). Examining the practice of generalist expertise: a qualitative study identifying constraints and solutions. JRSM Short Rep.

[16] Reeve J (2021). Avoiding harm: tackling problematic polypharmacy through strengthening expert generalist practice. Br J Clin Pharmacol.

[17] Hunter DJ (2009). Leading for health and wellbeing: the need for a new paradigm. J Public Health (Oxf).

[18] Rittel H, Webber M (1973). Dilemmas in general theory of planning. Policy Sci.

[19] Pawson R, Greenhalgh T, Harvey G, Walshe K (2005). Realist review - a new method of systematic review designed for complex policy interventions. J Health Serv Res Policy.

[20] Rycroft-Malone J, McCormack B, Hutchinson AM, DeCorby K, Bucknall TK, Kent B, et al. Realist synthesis: illustrating the method for implementation research. Implement Sci. 2012;7(1) Available from: https://pubmed.ncbi.nlm.nih.gov/22515663/. 10.1186/1748-5908-7-33 PMC3514310 22515663

[21] Wong G, Greenhalgh T, Westhorp G, Pawson R (2014). Development of methodological guidance, publication standards and training materials for realist and meta-narrative reviews: the RAMESES (Realist And Meta-narrative Evidence Syntheses – Evolving Standards) project. Health Serv Deliv Res.

[22] Home - ClinicalTrials.gov. Available from: https://clinicaltrials.gov/. Accessed 16 Oct 2018.

[23] Wong G (2018). Doing realist research.

[24] QSR International Pty Ltd (2018). NVivo qualitative data analysis software.

[25] Ross A, Gillett J. Forms of trust and polypharmacy among older adults. Ageing Soc. 2020:1–16. 10.1017/S0144686X20000483.

[26] Akinbolade O, Husband A, Forrest S, Todd A (2016). Deprescribing in advanced illness. Prog Palliat Care.

[27] Chen Z, Buonanno A (2017). Geriatric polypharmacy: two physicians’ personal perspectives. Clin Geriatr Med.

[28] Gillespie R, Mullan J, Harrison L (2018). Deprescribing for older adults in Australia: factors influencing GPs. Aust J Prim Health.

[29] McGrath K, Hajjar ER, Kumar C, Hwang C, Salzman B (2017). Deprescribing: a simple method for reducing polypharmacy. J Fam Pract.

[30] Ng WL, Tan MZW, Koh EYL, Tan NC (2017). Deprescribing: what are the views and factors influencing this concept among patients with chronic diseases in a developed Asian community?. Proc Singapore Healthc.

[31] Nadarajan K, Balakrishnan T, Yee ML, Soong JL (2018). The attitudes and beliefs of doctors towards deprescribing medications. Proc Singapore Healthc.

[32] Anderson K, Foster M, Freeman C, Luetsch K, Scott I (2017). Negotiating “unmeasurable harm and benefit”: perspectives of general practitioners and consultant pharmacists on deprescribing in the primary care setting. Qual Health Res.

[33] Bolmsjö BB, Palagyi A, Keay L, Potter J, Lindley RI (2016). Factors influencing deprescribing for residents in Advanced Care Facilities: insights from General Practitioners in Australia and Sweden. BMC Fam Pract.

[34] Clyne B, Cooper JA, Hughes CM, Fahey T, Smith SM, team O-S study (2016). “Potentially inappropriate or specifically appropriate?” Qualitative evaluation of general practitioners views on prescribing, polypharmacy and potentially inappropriate prescribing in older people. BMC Fam Pract.

[35] Drenth-van Maanen AC, Leendertse AJ, Jansen PAF, Knol W, Keijsers CJPW, Meulendijk MC (2018). The Systematic Tool to Reduce Inappropriate Prescribing (STRIP): combining implicit and explicit prescribing tools to improve appropriate prescribing. J Eval Clin Pract.

[36] Cenci C (2016). Narrative medicine and the personalisation of treatment for elderly patients. Eur J Intern Med.

[37] Schafer I, Kaduszkiewicz H, Mellert C, Loffler C, Mortsiefer A, Ernst A (2018). Narrative medicine-based intervention in primary care to reduce polypharmacy: results from the cluster-randomised controlled trial MultiCare AGENDA. BMJ Open.

[38] Baqir W, Barrett S, Desai N, Copeland R, Hughes J (2014). A clinico-ethical framework for multidisciplinary review of medication in nursing homes. BMJ Qual Improv Rep.

[39] Bokhof B, Junius-Walker U (2016). Reducing polypharmacy from the perspectives of general practitioners and older patients: a synthesis of qualitative studies. Drugs Aging.

[40] Cullinan S, Fleming A, O’Mahony D, Ryan C, O’Sullivan D, Gallagher P (2015). Doctors’ perspectives on the barriers to appropriate prescribing in older hospitalized patients: a qualitative study. Br J Clin Pharmacol.

[41] Frank C (2014). Deprescribing: a new word to guide medication review. CMAJ.

[42] Weir K, Nickel B, Naganathan V, Bonner C, McCaffery K, Carter SM (2018). Decision-making preferences and deprescribing: perspectives of older adults and companions about their medicines. J Gerontol B Psychol Sci Soc Sci.

[43] Moshinsky A, Bar-Hillel M. Loss aversion and status quo label bias. Soc Cogn. 28(2):191–204 Available from: https://psycnet.apa.org/record/2010-07534-004.

[44] Gnjidic D, Le Couteur DG, Hilmer SN. Discontinuing drug treatments. BMJ. 2014;349:g7013. 10.1136/bmj.g7013. 10.1136/bmj.g7013 25416561

[45] Ailabouni NJ, Nishtala PS, Mangin D, Tordoff JM (2016). General practitioners’ insight into deprescribing for the multimorbid older individual: a qualitative study. Int J Clin Pract.

[46] Cullinan S, Raae Hansen C, Byrne S, O’Mahony D, Kearney P, Sahm L (2017). Challenges of deprescribing in the multimorbid patient. Eur J Hosp Pharm.

[47] McCarthy C, Clyne B, Corrigan D, Boland F, Wallace E, Moriarty F (2017). Supporting prescribing in older people with multimorbidity and significant polypharmacy in primary care (SPPiRE): a cluster randomised controlled trial protocol and pilot. Implement Sci.

[48] Petersen AW, Shah AS, Simmons SF, Shotwell MS, Jacobsen JML (2018). Shed-MEDS: pilot of a patient-centered deprescribing framework reduces medications in hospitalized older adults being transferred to inpatient postacute care. Ther Adv Drug Saf.

[49] Manias E, Claydon-Platt K, McColl GJ, Bucknall TK, Brand CA (2007). Managing complex medication regimens: perspectives of consumers with osteoarthritis and healthcare professionals. Ann Pharmacother.

[50] Garfinkel D (2017). Overview of current and future research and clinical directions for drug discontinuation: psychological, traditional and professional obstacles to deprescribing. Eur J Hosp Pharm.

[51] Pruskowski J, Handler SM (2017). The DE-PHARM project: a pharmacistdriven deprescribing initiative in a nursing facility. Consult Pharm.

[52] Royal College of General Practitioners. About continuity of care. Available from: https://www.rcgp.org.uk/clinical-and-research/our-programmes/innovation/continuity-of-care/about-continuity-of-care.aspx. Accessed Dec 2020.

[53] Mainous AG, Baker R, Love MM, Gray DP, Gill JM (2001). Continuity of care and trust in one’s physician: evidence from primary care in the United States and the United Kingdom. Fam Med.

[54] NHS England. Medicines optimisation Available from: https://www.england.nhs.uk/medicines-2/medicines-optimisation/. Accessed Dec 2020.

[55] Reeve E, Thompson W, Farrell B (2017). Deprescribing: a narrative review of the evidence and practical recommendations for recognizing opportunities and taking action. Eur J Intern Med.

[56] Levina N, Vaast E (2005). The emergence of boundary spanning competence in practice: implications for implementation and use of information systems. MIS Q Manag Inf Syst.

[57] Kislov R, Hyde P, Mcdonald R, Manchester A (2017). New game, old rules? Mechanisms and consequences of legitimation in boundary spanning activities. Organ Stud.

[58] Dang BN, Westbrook RA, Njue SM, Giordano TP (2017). Building trust and rapport early in the new doctor-patient relationship: a longitudinal qualitative study. BMC Med Educ.

[59] Skirbekk H, Middelthon AL, Hjortdahl P, Finset A (2011). Mandates of trust in the doctor-patient relationship. Qual Health Res.

[60] Petrocchi S, Iannello P, Lecciso F, Levante A, Antonietti A, Schulz PJ (2019). Interpersonal trust in doctor-patient relation: evidence from dyadic analysis and association with quality of dyadic communication. Soc Sci Med.

[61] Halbesleben JRB, Neveu J-P, Westman M (2014). Getting to the “COR”: understanding the role of resources in conservation of resources theory. J Manage.

[62] Hupcey JE (1998). Clarifying the social support theory-research linkage. J Adv Nurs.

[63] Leahy-Warren P (2014). Social support theory. Theories guiding nursing research and practice: making nursing knowledge development explicit.

[64] Stewart M (2005). Reflections on the doctor-patient relationship: from evidence and experience. Br J Gen Pract.

[65] Laursen J, Kornholt J, Betzer C, Petersen TS, Christensen MB (2018). General practitioners’ barriers toward medication reviews in polymedicated multimorbid patients: how can a focus on the pharmacotherapy in an outpatient clinic support GPs?. Health Serv Res Manag Epidemiol.

[66] Crits-Christoph P, Rieger A, Gaines A, Beth M, Gibbons C (2019). Trust and respect in the patient-clinician relationship: preliminary development of a new scale. BMC Psychol.

[67] Birkhäuer J, Gaab J, Kossowsky J, Hasler S, Krummenacher P, Werner C, et al. Trust in the health care professional and health outcome: a meta-analysis. PLoS One. 2017;12(2) Available from: https://www.ncbi.nlm.nih.gov/pmc/articles/PMC5295692/. 10.1371/journal.pone.0170988 PMC5295692 28170443

[68] Ridge K (2021). Good for you, good for us, good for everybody: a plan to reduce overprescribing to make patient care better and safer, support the NHS, and reduce carbon emissions.

[69] National Institute for Health and Care Excellence (2015). Medicines optimisation: the safe and effective use of medicines to enable the best possible outcomes | Guidance | NICE.

[70] Reeve E (2020). Deprescribing tools: a review of the types of tools available to aid deprescribing in clinical practice. J Pharm Pract Res.

[71] Heaton J, Britten N, Krska J, Reeve J (2017). Person-centred medicines optimisation policy in England: an agenda for research on polypharmacy. Prim Health Care Res Dev.

[72] Reeve J (2021). Rethinking trust: the role of the Wise GP. Br J Gen Pract.

