# Correction: Optimising a person-centred approach to stopping medicines in older people with multimorbidity and polypharmacy using the DExTruS framework: a realist review

**DOI:** 10.1186/s12916-022-02611-x

**Published:** 2022-10-20

**Authors:** Amadea Turk, Geoffrey Wong, Kamal R. Mahtani, Michelle Maden, Ruaraidh Hill, Ed Ranson, Emma Wallace, Janet Krska, Dee Mangin, Richard Byng, Daniel Lasserson, Joanne Reeve

**Affiliations:** 1 Nuffield Department of Primary Care Health Sciences, Radcliffe Observatory Quarter, University of Oxford, Oxford, OX2 6GG UK; 2 Liverpool Reviews & Implementation Group, Institute of Population Health, University of Liverpool, Liverpool, L69 3BX UK; 3 Academy of Primary Care, Hull York Medical School, Allam Medical Building, University of Hull, Hull, HU6 7RX UK; 4 Department of General Practice RCSI University of Medicine and Health Sciences, Dublin 2, Ireland; 5 Medway School of Pharmacy, Universities of Greenwich and Kent, Chatham Maritime, Kent, ME4 4TB UK; 6 Department of Family Medicine, McMaster University, Hamilton, ON L8P 1H6 Canada; 7 Community and Primary Care Research Group, Peninsula Medical School, University of Plymouth, Plymouth, PL4 8AA UK; 8 Health Sciences, Warwick Medical School, University of Warwick, Coventry, CV4 7AL UK

**Correction: BMC Med 20, 297 2022**

**https://doi.org/10.1186/s12916-022-02475-1**

The original article [1] contained an error in the spelling of co-author, Michelle Maden’s name which has since been corrected.
