# Supplementary material for: Optimising a person-centred approach to stopping medicines in older people with multimorbidity and polypharmacy using the DExTruS framework: a realist review
Source: BMC Med. 2022 Aug 31;20:297. doi: 10.1186/s12916-022-02475-1 (PMC9429627; doi:10.1186/s12916-022-02475-1)
Supplement: Supplementary file 2 — Additional file 2. Table of included documents. [file 12916_2022_2475_MOESM2_ESM.docx]

## Supplementary Material – Table of documents included in the realist synthesis

| **Author** | **Year** | **Country** | **Title** | **Study Design/Methods** | **Sample/Setting** | **Objectives** |
| --- | --- | --- | --- | --- | --- | --- |
| Ahmad et al.^104^ | 2010 | Netherlands | Effect of medication review and cognitive behaviour treatment by community pharmacists of patients discharged from the hospital on drug related problems and compliance: design of a randomized controlled trial | Protocol for RCT | Patients over 60 discharged from general academic hospitals | To examine the effect of medication review and cognitive behaviour therapy of discharged patients by community pharmacists to minimize the occurrence of drug related problems |
| Ailabouni et al.^105^ | 2016 | New Zealand | General practitioners' insight into deprescribing for the multimorbid older individual: a qualitative study | Qualitative interview study | GPs prescribing for patients living in residential care | To explore GPs opinions and awareness of deprescribing in a hypothetical older multimorbid patient in residential care |
| Akinbolade et al.^106^ | 2016 | United Kingdom | Deprescribing in advanced illness | Literature review | Patients with advanced illness | To review reviews research on prescribing medicines to patients with advanced illness, focusing on the identification of the prevalence of inappropriate or unnecessary medicines to the initiation of the deprescribing process |
| Al Shemeili et al.^107^ | 2016 | United Arab Emirates | An exploration of health professionals’ experiences of medicines management in elderly, hospitalised patients in Abu Dhabi | Qualitative interview study | Healthcare professionals working in hospitals involved in medication management. The sample included, nurses, pharmacists and doctors | To describe and understand health professionals’ views and experiences of medicines management healthcare structures, processes and outcomes for elderly, hospitalised patients. |
| Altiner et al.^108^ | 2012 | Germany | Activating GENeral practitioners dialogue with patients on their Agenda (MultiCare AGENDA) study protocol for a cluster randomized controlled trial | Protocol for cluster RCT | General practice patients aged 65–84 years with at least 3 chronic conditions. Intervention | To investigate the efficacy of a complex multifaceted intervention aiming at increasing the quality of care of GPs for patients with multimorbidity through enhancing the doctor-patient-dialogue and identifying the patient’s agenda and needs. |
| Anderson et al.^109^ | 2017 | Australia | Negotiating “Unmeasurable Harm and Benefit”: Perspectives of General Practitioners and Consultant Pharmacists on Deprescribing in the Primary Care Setting | Qualitative focus group study | GPs and consultant pharmacists working in South East Queensland | To explore GPs’ and consultant pharmacists’ views about inappropriate polypharmacy, the reasoning they apply to deprescribing in primary care, and identify factors that support or inhibit this process. |
| Baqir et al.^110^ | 2014 | United Kingdom | A clinico-ethical framework for multidisciplinary review of medication in nursing homes | Quality improvement project | Pharmacists undertaking medication reviews with nursing home residents | To optimising medicines in care homes while involving all residents in decision making |
| Barnett et al.^18^ | 2016 | United Kingdom | Patient-centred management of polypharmacy: a process for practice | Review | Current UK literature around polypharmacy | To provide an overview of key guidance from the UK about polypharmacy and introduce a tool to support patient-centred practice |
| Bartlett Ellis and Welch^111^ | 2016 | USA | Medication-taking behaviours in chronic kidney disease with multiple chronic conditions: a meta-ethnographic synthesis of qualitative studies | Systematic review- meta-ethnography | Literature on medication-taking behaviour in chronic kidney disease | To identify behaviours associated with taking medications and medication adherence reported in qualitative studies of adults with chronic kidney disease and coexisting multiple chronic conditions. |
| Beuscart et al. ^93^ | 2018 | Belgium | Development of a core outcome set for medication review in older patients with multimorbidity and polypharmacy: a study protocol | Mixed methods – systematic review, semi-structured interviews, Delphi survey | Older patients with multimorbidity and polypharmacy | To describe a method that could be used to develop a core outcome set for use in trials of older patients with multimorbidity. |
| Bokhof and Walker^112^ | 2016 | Germany | Reducing Polypharmacy from the Perspectives of General  Practitioners and Older Patients: A Synthesis of Qualitative  Studies | Systematic review- meta-ethnography | General practitioners and older patients | To synthesize qualitative studies exploring the perspectives and experiences of general practitioners (GPs) and older patients in reducing polypharmacy and to discover approaches already being practiced. |
| Bolmsjo et al.^113^ | 2016 | Sweden and Australia | Factors influencing deprescribing for residents in Advanced Care Facilities: insights from General Practitioners in Australia and Sweden | Qualitative Synthesis of two interview studies | General practitioners serving patients in long-term care facilities | To compare and contrast behavioural factors influencing the prescribing practices of GPs providing care in advanced care facilities in two different countries; to review health policy and aged care facility systems in each setting for their potential impact on the prescribing of medications; based on these finding provide recommendations |
| Cashman et al.^114^ | 2010 | United Kingdom | The treatment of co-morbidities in older patients with metastatic cancer | Review of medical records and patient interviews | Patients with metastatic cancer | To determine whether older patients with metastatic cancer continue to take medications for the treatment of pre-existing comorbidities after the diagnosis of metastatic disease |
| Cenci^115^ | 2016 | Italy | Narrative medicine and the personalisation of treatment for elderly patients | Literature review | Patients with multimorbidity and polypharmacy | To provide an overview of how narrative medicine can promote the development of a systematic, integrated and multi-disciplinary approach to older patients |
| Centeno and Fullerton^116^ | 2016 | USA | Got pills? A pharmacist’s impact on chronic disease and older adults in transitions of care | Conference abstract | Quality improvement project | To assess the impact of medication reconciliation by clinical pharmacist on patient outcomes during transitions of care. |
| Chen and Buonanno^117^ | 2017 | USA | Geriatric Polypharmacy Two Physicians’ Personal Perspectives | Opinion piece | Two clinicians discussing experiences of managing polypharmacy | To discuss geriatric polypharmacy from 2 practitioners’ viewpoints |
| Cheraghi-Sohi et al.^118^ | 2015 | United Kingdom | The influence of personal communities on the self-management of medication taking: A wider exploration of medicine work | Qualitative interview study | Patients with long term conditions | To explicate: the nature of the work that people with multiple LTCs, and their network members do, in attempting to take their medications on a daily basis, the division of labour amongst these members and when and why network members become involved in that work. |
| Christensen et al. 2017^119^ | 2017 | Denmark | Physicians’ Non-Uniform Approach to Prescribing Drugs to Older Patients – A Qualitative Study | Qualitative interview study | Medical specialists working with older patients | To explore physicians’ approach to prescribe drugs to older patients, including identifying the drugs that physicians perceive to be risk drugs for older patients and comparing them with established lists of potentially inappropriate medications. |
| Cimmino and Pisano^120^ | 2016 | USA | A Patient’s Last Wish at the End of Life | Case study | Patients at the end of life | A case study discussing managing polypharmacy at the end of life |
| Clyne et al.^121^ | 2016 | Ireland | ‘Potentially inappropriate or specifically appropriate?’ Qualitative evaluation of general practitioners views on prescribing, polypharmacy and potentially inappropriate prescribing in older people | Qualitative Interview Study | GPs participating in a randomised controlled trial (RCT) of an intervention to decrease potentially inappropriate prescribing in older patients (≥70 years) in Ireland | To explore GP perspectives regarding prescribing and potentially inappropriate prescribing in older primary care patients. |
| Cullinan et al. ^122^ | 2017 | Ireland | Challenges of deprescribing in the multimorbid patient | Literature review | Literature on challenges to deprescribing in patients with multimorbidity | To highlight some of the potential reasons for this lack of deprescribing and the challenges to discontinuing drugs for these patients. |
| Cullinan et al. ^123^ | 2015 | Ireland | Doctors’ perspectives on the barriers to appropriate prescribing in older hospitalized patients: a qualitative study | Qualitative interview study | Hospital doctors prescribing for older people | (i) To identify hospital doctors’ perceptions as to why PIP occurs, (ii) to identify the barriers to addressing the issues identiﬁed and (iii) to determine which intervention types would be best suited to improving prescribing. |
| Djatche et al.^124^ | 2017 | Italy | How confident are physicians in deprescribing for the elderly and what barriers prevent deprescribing? | Survey | Primary Care physicians | to assess the perceptions of primary care physicians on deprescribing for elderly patients and potential barriers to deprescribing that physicians experience in the Local Health Authority (LHA) of Parma, Emilia Romagna, Italy. |
| Drenth‐van Maanen et al. ^125^ | 2017 | Netherlands | The Systematic Tool to Reduce Inappropriate Prescribing (STRIP): Combining implicit and explicit prescribing tools to improve appropriate prescribing | Presentation of a prescribing tool | n/a | To describe the Systematic Tool to Reduce Inappropriate Prescribing (STRIP) and its ability to identify potentially inappropriate prescribing. |
| Duncan et al.^126^ | 2017 | United Kingdom | Deprescribing: a primary care perspective | Literature review | Literature on deprescribing and polypharmacy in primary care | To (i) describe  trends in polypharmacy and explanations for why it is increasing; (ii) outline the harms associated with overtreatment; (iii) outline the rationale for deprescribing and different approaches to deprescribing within general practice, including the role of the pharmacist; (iv) outline the barriers and enablers to deprescribing; and (v) make recommendations for future practice. |
| Edelman et al.^127^ | 2019 | Netherlands | Patients’ Attitudes Towards Deprescribing Alpha‑Blockers and Their Willingness to Participate in a Discontinuation Trial | Questionnaire | Men aged 30 years and older with lower urinary tract symptoms, who were first prescribed an alpha-blocker in 2015 or 2016 | To gain insights into the attitudes of men with lower urinary tract symptoms towards deprescribing alpha-blockers and to assess their willingness to participate in a planned discontinuation trial. |
| Elliott et al.^128^ | 2007 | USA | Strategies for Coping in a Complex World: Adherence Behavior Among Older Adults with Chronic Illness | Qualitative interview study | Older adults taking multiple medications | To explore how older adults with multiple illnesses make choices about medicines. |
| Frank^129^ | 2014 | Canada | Deprescribing: a new word to guide medication review | Commentary | n/a | To describe deprescribing |
| Fried et al.^70^ | 2017 | USA | Effect of the Tool to Reduce Inappropriate Medications (TRIM) on Medication Communication and Deprescribing | Randomised control trial | 128 Veterans age 65 years and older prescribed  7 medications, randomized to  receipt of TRIM or usual care | To examine the effect of TRIM (Tool to Reduce Inappropriate Medications), a web tool linking the electronic health record (EHR) to a clinical decision support system, on medication communication and prescribing. |
| Garfinkel^130^ | 2017 | Israel | Overview of current and future research and clinical directions for drug discontinuation: psychological, traditional and professional obstacles to deprescribing | Literature review/ commentary | n/a | To provide an overview of and future research and clinical directions for drug discontinuation. |
| Gaup and Halvorsen^131^ | 2015 | Norway | Physicians’ experiences with NORGEP criteria and the use of inappropriate medication in elderly patients in nursing home and home care service | Conference abstract for qualitative interview study | Nursing home physicians and general practitioners | To investigate how nursing home physicians and general practitioners’ cope with inappropriate prescribing, their own experiences of using NORGEP criteria in clinical work, and how inappropriate prescribing could be reduced. |
| Geijteman et al.^132^ | 2018 | Netherlands | Medication Discontinuation at the End of Life:  A Questionnaire Study on Physicians’ Experiences and Opinions | Questionnaire | General practitioners and clinical specialists working in three regions in the Netherlands | To explore physicians’ opinions and experiences regarding medication discontinuation during the last phase of life, and to identify factors inﬂuencing the continuation of potentially inappropriate medications. |
| Gillespie et al. ^133^ | 2018 | Australia | Deprescribing for older adults in Australia: factors inﬂuencing GPs | Survey | General practitioners | To explore factors that inﬂuence deprescribing among Australian GPs using a new 21-item survey to measure GP attitudes and practices. |
| Gnjidic et al.^134^ | 2012 | Australia | Deprescribing Trials: Methods to Reduce  Polypharmacy and the Impact on Prescribing and Clinical Outcomes | Literature review | Literature on interventions designed to reduce polypharmacy on prescribing and clinical outcomes | To highlight the evidence for the impact of various types of interventions designed to reduce polypharmacy on prescribing and clinical outcomes in older adults from community, nursing home, and hospital settings. |
| Gnjidic et al ^135^ | 2014 | Australia | Discontinuing drug treatments: We need better evidence to guide deprescribing | Commentary | n/a | To describe the evidence base for deprescribing |
| Goncalves ^136^ | 2018 | Portugal | Deprescription in Advanced Cancer Patients | Literature review | n/a | To describe deprescribing in cancer patients and propose a six-step method for deprescription |
| Hardy and Hilmer^137^ | 2011 | Australia | Deprescribing in the Last Year of Life | Literature review | n/a | To provide an algorithm to guide safe, rational deprescribing for patients who are believed to be in their last year of life. |
| Harriman et al.^138^ | 2015 | Canada | Deprescribing medication for frail elderly patients in nursing homes: A survey of Vancouver family physicians | Survey | Family physicians | To understand the beliefs and approaches of experienced family physicians (FPs) to help identify ways to improve current practices and reduce polypharmacy among frail elderly patients. |
| Hasler et al.^139^ | 2015 | Switzerland | Effect of a patient-centered drug review on polypharmacy in primary care patients: study protocol for a cluster-randomized controlled trial | Protocol for a cluster-randomized controlled trial | Primary Care physicians | To determine whether a patient-centered systematic review leads to more appropriate medication use in patients without negatively affecting quality of life and the course of the disease. |
| Heaton et al.^32^ | 2017 | United Kingdom | Person-centred medicines optimisation policy in England: an agenda for research on polypharmacy | Review of policy – documentary analysis of reports on medicines optimisation | Policy reports on medicines optimisation published by the Royal Pharmaceutical Society (RPS), The King’s Fund and National Institute for Health and Social Care Excellence since 2013 | To examine how patient perspectives and person-centred care values have been represented in documents on medicines optimisation policy in England. |
| Hernandez^140^ | 2017 | USA | Medication management in the older adult: A narrative  exploration | Qualitative interview study | Nurse practitioners caring for older adults | To characterize the meaning nurse practitioners (NPs) ascribed to personal experiences providing care to older adults who take multiple medications to manage complex conditions. |
| Hilmer et al.^141^ | 2012 | Australia | Thinking through the medication list:  Appropriate prescribing and deprescribing in robust and frail older patients | Literature review | n/a | To provide an ethically sound, evidence based discussion of the benefits and harms of medications commonly used in primary care among older patients. |
| Howland ^142^ | 2012 | USA | Questions to Ask When Selecting Medication | Commentary/opinion piece | n/a | To explore eight questions that should be considered when selecting medication for a patient |
| Jager et al.^143^ | 2015 | Germany | Medication Lists and Brown Bag Reviews: Potential Positive and Negative Impacts on Patients Beliefs about Their Medicine | Cross-sectional study with survey | Patients aged over 50 taking more than 4 drugs enrolled into the “Polypharmacy in Multimorbid Patients study | To explore whether patients’ use of a medication list is associated with their beliefs about their medicine and their memory of structured medication counselling. |
| Jansen et al.^144^ | 2017 | Australia | General Practitioners’ Decision Making about Primary Prevention of Cardiovascular Disease in Older Adults: A Qualitative Study | Qualitative interview study | General practitioners | To explore GPs’ decision making about primary CVD prevention in patients aged 75 years and older. |
| Jones^145^ | 1997 | USA | Decreasing polypharmacy in clients most at risk | Commentary/opinion piece | n/a | To give an overview of decreasing polypharmacy |
| Kaufman et al.^146^ | 2017 | United Kingdom | Considering patient experience and evidence-based choice of medicines  in medicines optimisation | Continuing professional development (CPD) module | n/a | To discuss the challenges of medicines optimisation, a patient-focused approach to supporting patients to gain maximum benefit from their medicines |
| Knowles et al.^147^ | 2017 | United Kingdom | Empowering people to help speak up about safety in primary care: Using co-design to involve patients and professionals in developing new interventions for patients with multimorbidity | Accelerated experience- based co-design and the future workshop approach | Healthcare professionals and patients | To explore whether coproduction methodologies could enhance intervention development and provide a mechanism to translate available evidence into patientcentred intervention proposals for multimorbidity and safety. |
| Koberlein et al.^148^ | 2013 | Germany | General practitioners’ views on polypharmacy and its consequences for patient health care | Study protocol for a Retrospective cross sectional study using mixed methods | General practitioners and patients | To detect the status quo of the health care situation in Saxony’s general practices for multimorbid patients receiving multiple medications. |
| Komagamine et al.^73^ | 2018 | Japan | Characteristics of elderly patients with polypharmacy who refuse to participate in an in-hospital deprescribing intervention: a retrospective cross-sectional study | Retrospective cross-sectional study | Patients aged 65 years or older who reported the use of five or more medications upon admission to the orthopedic ward from January 2015 to December 2016 and who were approached by a pharmacist for polypharmacy screening | To evaluate the prevalence of potentially inappropriate medication (PIM) use in elderly patients accepting and refusing a deprescribing intervention and to investigate factors associated with deprescribing refusal. |
| Krska^149^ | 2018 | United Kingdom | Factoring in frailty when optimising medication | Opinion piece/commentary | n/a | To give advice to help identify frailty and adopt a patient- centred approach to medicines optimisation. |
| Krska et al.^150^ | 2014 | United Kingdom | Measuring the impact of long-term medicines use from the patient perspective | Commentary | n/a | To discuss measuring the impact of long-term medicines use from the patient perspective |
| Kuruvilla et al.^151^ | 2018 | Australia | Medication management for community palliative care patients and the role of a specialist palliative care pharmacist: A qualitative exploration of consumer and health care professional perspectives | Qualitative focus group study | Palliative care consumers and clinicians specifically patients, caregivers, physicians, nurses and pharmacists. | To explore the perspectives of stakeholders about the gaps in the current model of community palliative care services in relation to medication management and to assess their opinions pertaining to the role of a specialist palliative care pharmacist in addressing some of those gaps. |
| Laursen et al.^152^ | 2018 | Denmark | General Practitioners’ Barriers Toward Medication Reviews in Polymedicated Multimorbid Patients: How can a Focus on the Pharmacotherapy in an Outpatient Clinic Support GPs? | Qualitative interview study | General practitioners | To explore whether general practitioners (GPs) experienced barriers toward medication reviews in polymedicated, multimorbid patients, and how a clinical pharmacologist with a focus on pharmacotherapy can support the GPs in an outpatient clinic. |
| Maidment et al.^153^ | 2017 | United Kingdom | Developing a framework for a novel multidisciplinary, multi-agency intervention(s), to improve medication management in community-dwelling older people on complex medication regimens (MEMORABLE)––a realist synthesis | Protocol for a realist synthesis | Literature on medication management in older people on complex medication regimes residing in the community | To understand how, why, for whom and in what context interventions, to improve medication management in older people on complex medication regimes residing in the community, work. |
| Mangin et al.^14^ | 2018 | Canada | International Group for Reducing Inappropriate Medication Use & Polypharmacy (IGRIMUP): Position Statement and 10 Recommendations for Action | Opinion piece | n/a | To present the ﬁrst position statement of IGRIMUP (International Group for Reducing Inappropriate Medication Use & Polypharmacy) on the international co-operative effort, and recommendations for actions needed to prevent and counter IMUP and its drivers globally. |
| Manias et al. ^154^ | 2007 | Australia | Managing Complex Medication Regimens: Perspectives of Consumers with Osteoarthritis and Healthcare Professionals | Qualitative focus group and interview study | Patients and healthcare professionals | To examine medication management for osteoarthritis and other chronic conditions from the perspectives of community-dwelling consumers and healthcare professionals, using a qualitative approach. |
| Mantelli et al. ^155^ | 2018 | Switzerland | How general practitioners would deprescribe in frail oldest-old with polypharmacy — the LESS study | Survey | General practitioners | To determine whether, how, and why Swiss GPs deprescribe for the oldest-old (> 80-years) with multimorbidity and polypharmacy |
| Marengoni et al.^156^ | 2015 | Italy | Best Practices for Drug Prescribing in Older Adults: A Call for Action | Opinion piece | n/a | To propose a multicomponent intervention with the goal of achieving the best-tailored pharmacotherapy |
| McCarthy et al.^74^ | 2017 | Ireland | Supporting prescribing in older people with multimorbidity and significant polypharmacy in primary care (SPPiRE): a cluster randomised controlled trial protocol and pilot | Protocol for a cluster randomised controlled trial | General practice patients (aged 65 and over with 15 or more prescribed medications) and general practitioners | To assess the effectiveness of a complex intervention designed to support general practitioners (GPs) to reduce potentially inappropriate prescribing and consider deprescribing in older people with multimorbidity and significant polypharmacy in Irish primary care |
| McGrath et al.^157^ | 2017 | USA | Deprescribing: A simple method for reducing polypharmacy | Commentary/opinion piece using a case study | n/a | To present a four step plan to aid the safe deprescribing in older adults |
| Mc Namara et al.^158^ | 2017 | Australia | Health professional perspectives on the management of multimorbidity and polypharmacy for older patients in Australia | Qualitative interview study | Healthcare professionals including nurses, doctors, dentists, pharmacists, physiotherapists working in a range of settings | To explore current approaches to multimorbidity management, and perceived barriers and enablers to deliver appropriate medications management for community-dwelling patients with multimorbidity and polypharmacy, from a broad range of healthcare professional (HCP) perspectives in Australia |
| Modig et al.^159^ | 2009 | Sweden | Frail elderly patients in primary care—their medication knowledge and beliefs about prescribed medicines | Questionnaire | Patients aged 65 years and above with multiple illnesses | To describe elderly patients’ knowledge about and attitudes towards their medicines in Swedish primary care. |
| Molokhia and Majeed^161^ | 2017 | United Kingdom | Current and future perspectives on the management of polypharmacy | Opinion piece | n/a | To review trends in polypharmacy and how clinicians can try to ensure they maximise the benefits of prescribing and minimise the associated complications; particularly in the increasing number of frail, elderly patients that physicians are now seeing in health systems across the world. |
| Mudge et al.^160^ | 2016 | Australia | Impact of a pilot multidisciplinary clinic for frequent attending elderly patients on deprescribing | Retrospective study | Patients with frequent medical admissions | To examine the impact of the THRIVE model on medication count, tablet load and potentially inappropriate medicines (PIMs). |
| Nadarajan et al.^162^ | 2018 | Singapore | The attitudes and beliefs of doctors towards deprescribing medications | Survey | Hospital doctors | To explore the attitudes and beliefs of deprescribing medications among doctors in the Department of Internal Medicine (DIM) in Singapore General Hospital (SGH), and second, to see if differences exist among junior and senior doctors in their attitudes towards deprescribing. |
| Naughton and Hayes^163^ | 2016 | United Kingdom | Deprescribing in older adults: a new concept for nurses in administering medicines and as prescribers of medicine | Literature review | n/a | To examine the context of deprescribing from the perspective of nurses in medicines administration and prescribing practices and outlines the nature of the nursing contribution to this emerging topic |
| Ng et al.^164^ | 2017 | Singapore | Deprescribing: What are the views and factors influencing this concept among patients with chronic diseases in a developed Asian community? | A cross-sectional study using the validated Patients’ Attitudes Towards Deprescribing (PATD) questionnaire | Patients on regular follow-up at the clinics for chronic disease management and with at least five regular prescription medications | To elucidate patients’ attitudes towards the number of medications they were taking and identify factors that might influence acceptance of deprescription. |
| Nixon and Vendelo^165^ | 2016 | Denmark | General practitioners’ decisions about discontinuation of  medication: an explorative study | Qualitative interviews and observations | General practitioners | To investigate how general practitioners’ (GPs) decisions about discontinuation of medication are influenced by their institutional context. |
| Drug and Therapeutics Bulletin^166^ | 2016 | United Kingdom | Frailty, polypharmacy and deprescribing | Commentary | n/a | To provide an overview of frailty, polypharmacy and deprescribing |
| Oboh and Qadir^167^ | 2017 | United Kingdom | Deprescribing and managing polypharmacy in frail older people: a patient-centred approach in the real world | Case report | a 73-year-old diabetic man taking multiple medication, with gastrointestinal (GI) and pain symptoms as well as poor adherence to medicines. | To describe a pharmacist-led, patient-centred approach to deprescribing in a 73-year-old diabetic man taking multiple medication, with gastrointestinal (GI) and pain symptoms as well as poor adherence to medicines. |
| O’Brien^168^ | 2011 | Canada | Withdrawing medication Managing medical comorbidities near the end of life | Case report | 67-year-old woman with a long smoking history, presents to you with dyspnea, cough with hemoptysis, fatigue, and weight loss, as well as low back and left hip pain | To discuss withdrawing medication in a patient with multimorbidity near the end of life |
| Ouellet et al.^169^ | 2018 | USA | Principle of rational prescribing and deprescribing in older adults with multiple chronic conditions | Literature review | n/a | To provide a reasoned approach to medication prescribing and deprescribing decisions for older adults with multiple chronic conditions, which aims to achieve clinical outcomes that matter most to each individual patient. |
| Page et al.^170^ | 2016 | Australia | Deprescribing in older people | Narrative literature review | Literature on deprescribing | To describe the genesis of deprescribing as an increasingly accepted medical and pharmaceutical intervention. It also provides an overview of deprescribing. |
| Palagyi et al.^171^ | 2016 | Australia | Barricades and brickwalls – a qualitative study exploring perceptions of medication use and deprescribing in long-term care | Qualitative focus group and interview study | GPs, staff members, residents and their relatives within long term care facilities (LTCF) | To report the perceptions of medication use and the concept of deprescribing for LTCF residents, as identified by the RELEASE study participants. The application of these findings to informing the development of deprescribing initiatives within the aged care sector is discussed. RELEASE aims to improve our understanding of the attitudes towards medication reduction held by the frail elderly in residential care. |
| Petersen et al.^173^ | 2018 | USA | Shed-MEDS: pilot of a patient-centred deprescribing framework reduces medications in hospitalized older adults being transferred to inpatient post-acute care | Cross-sectional study | 40 Medicare-eligible, hospitalized patients with at least five prescribed medications. | To describe a hospital-based, patient-centered deprescribing protocol (Shed-MEDS) and report pilot results. |
| Pitkala et al.^174^ | 2016 | Finland | Herbal medications and other dietary supplements. A clinical review for physicians caring for older people | Literature review | Literature regarding older people’s use of dietary supplements with special reference to polypharmacy. | To conduct a literature review on clinical considerations associated with dietary supplement use, focusing on benefits and harms, motivations for use and contribution to polypharmacy among older people |
| Le Couteur^175^ | 2016 | Australia | Polypharmacy in older people: When should you deprescribe? | Opinion piece/commentary | n/a | To describe the challenges of managing multimorbidity and polypharmacy and present an individualised person-centred approach that takes into account multimorbidity |
| Pruskowski and Handler^176^ | 2017 | USA | The DE-PHARM Project: A Pharmacist Driven Deprescribing Initiative in a Nursing Facility | Quality improvement project | Residents in a nursing facility | To reduce the number of potentially inappropriate medications via accepted recommendations from the clinical pharmacist to the primary team. |
| Reeve et al.^177^ | 2014 | Australia | Review of deprescribing processes and development of an evidence-based, patient-centred deprescribing process | Literature review | n/a | To describe the development of a patient-centred deprescribing process |
| Reeve et al.^178^ | 2015 | Australia | Barriers to Optimising Prescribing and Deprescribing in Older Adults with Dementia: A Narrative Review | Narrative review of the literature | Literature on optimising medications in older adults with dementia | To explore barriers to optimising prescribing and deprescribing of medication as the goal of care shifts from prolonging life to optimising quality of life. |
| Reeve et al.^6^ | 2018 | United Kingdom | Identifying enablers and barriers to individually tailored prescribing: a survey of healthcare professionals in the UK | Survey | 419 health professionals across the UK | To examine health professionals’ perceptions of enablers and barriers to delivering individually tailored prescribing. |
| Rieckert et al.^179^ | 2018 | Germany | Reduction of inappropriate medication in older populations by electronic decision support (the PRIMA-eDS study): a qualitative study of practical implementation in primary care | Qualitative interview study | General practitioners belonging to the intervention group of the PRIMA-eDS study | To examine how GPs experienced the use of the PRIMA-eDS tool, how GPs adopted the recommendations provided by the CMR, and explores GPs’ ideas on the future implementation of the tool. |
| Rigby^180^ | 2013 | Australia | Interview crucial to HMR success | Opinion piece/commentary | n/a | To discuss the importance of interviews in home medications review |
| Rodriguez Perez^181^ | 2015 | Spain | Deprescribing in patients with multimorbidity: A necessary process | Opinion piece/commentary | n/a | To discuss the importance of deprescribing in patients with multimorbidity |
| Rose et al.^182^ | 2019 | Germany | Patient selection and general practitioners' perception of collaboration in medication review | Qualitative interview study | General Practitioners | To gain information on patient selection for a MR by general practitioners (GPs). GP selection was compared to objective selection criteria on identifying patients, who would beneﬁt from a MR the most. A secondary objective of this study was to get insight into GPs perception on interprofessional collaboration with pharmacists. |
| Ross and Gillett^183^ | 2020 | Canada | Confronting Medicine’s Dichotomies: Older Adults’ Use of Interpretative Repertoires in Negotiating the Paradoxes of Polypharmacy and Deprescribing | Qualitative interview study | Older adults aged over 70 taking part in the TAPER trial | To identify the medication paradoxes experienced by older adults taking multiple medications and describe the work that older adults do to bring them to resolution. |
| Ross and Gillet^184^ | 2020 | Canada | “At 80 I Know Myself”: Embodied Learning and Older Adults’ Experiences of Polypharmacy and Perceptions of Deprescribing | Qualitative interview study | Older adults aged over 70 taking part in the TAPER trial | To examine the forms of expertise that inform older adults’ decisions about how to use medications given concerns over polypharmacy and a clinical focus on deprescribing. |
| Ross and Gillett^185^ | 2020 | Canada | Forms of trust and polypharmacy among older adults | Qualitative interview study | Older adults aged over 70 taking part in the TAPER trial | To examine how older adults make decisions about their medications through interconnected axes of trust that operate across social networks. |
| Ryan and Hill^186^ | 2016 | Australia | Making rational choices about how best to support consumers’ use of medicines: a perspective review | Literature review | n/a | To present perspectives on how to support consumers’ use of medicines |
| Schäfer et al.^187^ | 2017 | Germany | Narrative medicine-based intervention  in primary care to reduce polypharmacy: results from the cluster-randomised controlled trial MultiCare AGENDA | Two-arm cluster-randomised controlled trial | 604 patients 65 to 84 years of age with at least three chronic conditions in general practice | To determine if patient-centred communication leads to a reduction of the number of medications taken without reducing health-related quality of life. |
| Schöpf et al.^188^ | 2018 | Germany | Elderly patients’ and GPs’ perspectives of patient–GP communication concerning polypharmacy: a qualitative interview study | Qualitative interview study | Patients of at least 65 years old with polypharmacy (⩾5 medications) and their GPs in a German Primary Healthcare Centre | To explore elderly patients’ and general practitioners’ (GPs’) perceptions of communication about polypharmacy, medication safety and approaches for empowerment. |
| Schuling et al.^189^ | 2012 | Netherlands | Deprescribing medication in very elderly patients with multimorbidity: the view of Dutch GPs. A qualitative study | Qualitative focus group study | GPs with a minimum of five years experience and active as GP trainers. | To explore how experienced GPs feel about deprescribing medication in older patients with multimorbidity and to what extent they involve patients in these decisions. |
| Scott et al.^190^ | 2013 | Australia | Deciding when to stop: towards evidence-based deprescribing of drugs in older populations | Opinion piece/commentary | n/a | To describe the evidence base for a structured approach to deprescribing and explore the barriers that exist in routine practice. |
| Sheppard et al.^191^ | 2018 | United Kingdom | OPtimising Treatment for MIld Systolic hypertension in the Elderly  (OPTiMISE): protocol for a randomised controlled non-inferiority trial | protocol for a randomised controlled non-inferiority trial | Participants aged ≥80 years, with systolic blood pressure <150 mm Hg and receiving ≥2 antihypertensive medications. | To examine whether antihypertensive medication reduction is possible in older patients without significant changes in blood pressure control at follow-up. |
| Sinnige et al.^192^ | 2016 | Netherlands | Medication management strategy for older people with polypharmacy in general practice: a qualitative study on prescribing behaviour in primary care | Qualitative focus group study | Dutch GPs | To gain insight into GPs’ medication management strategies for patients with polypharmacy, and to explore the GPs’ perspectives and needs on decisionmaking support to facilitate this medication management. |
| Sinnott et al.^193^ | 2015 | Ireland | What to give the patient who has everything? A qualitative study of prescribing for multimorbidity in primary care | Qualitative interview study | Irish GPs | To explore how GPs make decisions when prescribing for multimorbid patients, with a view to informing intervention design. |
| Sinnott et al.^194^ | 2015 | Ireland | Improving medication management in multimorbidity: development of the MultimorbiditY COllaborative Medication Review And DEcision Making (MY COMRADE) intervention using the Behaviour Change Wheel | Development of a medication review decision making tool- systematic review and qualitative study with GPs | GPs | To describe how we have used results from a review of previous research, original research of our own and the Behaviour Change Wheel to develop an intervention to improve medication management in multimorbidity by general practitioners (GPs), within the overarching UK Medical Research Council guidance on complex interventions |
| Sinnott et al.^195^ | 2017 | Irelands | Improving medication management for patients with multimorbidity in primary care: a qualitative feasibility study of the MY COMRADE implementation intervention | non-randomised feasibility study using a qualitative framework approach | GPs attending continuing professional development meetings (CPD) in southwest Ireland | To assess the feasibility and acceptability of MY COMRADE by GPs. |
| St Peter^196^ | 2015 | USA | Management of Polypharmacy in Dialysis Patients | Opinion piece/commentary | n/a | To discuss the management of polypharmacy in dialysis patients |
| Steinman and Hanlon^197^ | 2010 | USA | Managing medications in clinically complex elders: “There’s got to be a happy medium” | Case study | 84-year-old man with dementia with a history of atrial fibrillation, diabetes mellitus, hypertension, hyperlipidemia, chronic kidney disease (estimated creatinine clearance of 42ml/min), and gastritis and gastroesophageal reflux disease. | To describe a typical case of an older patient taking multiple medications and summarizes the evidence-based literature about improving medication use and withdrawing specific drugs and drug classes. To present a systematic approach for how health professionals can assess and improve medication regimens. |
| Straßner et al.^198^ | 2018 | Germany | German healthcare professionals' perspective on implementing recommendations about polypharmacy in general practice: A qualitative study | Qualitative interview and focus group study | 24 general practitioners (GPs), 4 other medical specialists, 1 pharmacist, 3 nurses and 6 medical assistants as well as 2 mixed focus groups with 17 professionals | T o identify determinants (hindering and facilitating factors) for the implementation of the recommendations in general practice. |
| Sun et al.^199^ | 2019 | Canada | Exploration of home care nurse’s experiences in deprescribing of medications: a qualitative descriptive study | Qualitative focus group study | 11 home care nurses | To explore the barriers and enablers of deprescribing from the perspectives of home care nurses, as well as to conduct a scalability assessment of an educational plan to address the learning needs of home care nurses about deprescribing. |
| Thomas and Killbey^200^ | 2011 | United Kingdom | Patients with one or more long-term conditions often take multiple prescribed medications. A joint approach to drug management improved quality and cut costs | Pilot project of multidisciplinary reviews for patients with complex needs | four patients with complex needs | To improve the quality of care for patients receiving multiple prescribed medicines for one or more long-term conditions, using a holistic, evidence-based approach. |
| Townsend et al.^201^ | 2003 | United Kingdom | Managing multiple morbidity in mid-life: a qualitative study of attitudes to drug use | Qualitative interview study | 23 men and women aged about 50 years with four or more chronic illnesses. | To examine attitudes towards drug use among middle aged respondents with high levels of chronic morbidity |
| Turner et al.^202^ | 2016 | Australia | What factors are important for deprescribing in Australian long-term care facilities? Perspectives of residents and health professionals | Qualitative research using nominal group technique | 11 residents/representatives, 19 GPs, 12 nurses and 14 pharmacists participated across six separate groups. | To use nominal group technique (NGT) to generate then rank factors that general medical practitioners (GPs), nurses, pharmacists and residents or their representatives perceive are most important when deciding whether or not to deprescribe medications |
| Turner et al. ^203^ | 2017 | Australia | Is my older cancer patient on too many medications? | Commentary/opinion piece | n/a | To present six step process for deprescribing in older patients with cancer |
| Twigg et al.^204^ | 2017 | United Kingdom | The UK Pharmacy Care Plan service: Description, recruitment and initial views on a new community pharmacy intervention | Mixed methods using questionnaires and interviews | Pharmacists and patients | To describe the initial findings from the set up and delivery of a novel community pharmacybased person-centred service. |
| Uhl et al.^205^ | 2018 | Germany | Patient-perceived barriers and facilitators to the implementation of a medication review in primary care: a qualitative thematic analysis | Qualitative interview study | 31 patients (age ≥ 60 years, ≥3 chronic diseases, taking ≥5 drugs) | To gain insight into patient-perceived barriers and facilitators to the implementation of medication review. |
| Van Middelaar et al.^206^ | 2018 | Netherlands | Prescribing and deprescribing antihypertensive medication in older people by Dutch general practitioners: a qualitative study | Qualitative interview study | 15 GPs | To explore general practitioners’ (GPs) routines and considerations on (de)prescribing antihypertensive medication (AHM) in older patients, their judgement on usability of the current guideline and needs for future support. |
| Van Summeren et al.^83^ | 2017 | Netherlands | Outcome prioritisation tool for medication review in older patients with multimorbidity: a pilot study in general practice | Mixed methods descriptive study | older patients with multimorbidity (aged ≥69 years) with polypharmacy (five or more chronic medications) from the practices of 14 GPs | To determine proposed and observed medication changes when using an outcome prioritisation tool (OPT) during a medication review in general practice. |
| Vandermause et al. ^207^ | 2016 | USA | Preserving Self: Medication-Taking Practices and Preferences of Older Adults With Multiple Chronic Medical Conditions | Qualitative study using interviews and assessment of diaries | 27 participants with multiple chronic conditions | To examine the experiences of older adults with multiple chronic medical conditions when a new medication was added to their existing multiple medication regimen. |
| Voigt et al.^208^ | 2016 | Germany | Why do family doctors prescribe potentially inappropriate medication to elderly patients? | Mixed methods using 10 semi-standardized content analysis of patients’ records, 2) qualitative interviews with FPs using a) open questions and b) selected patient-specific case vignettes and 3) qualitative interviews with FPs’ medical assistants. | Patients and family physicians | 1) to give an overview of rates of PIM prescription in our study sample of elderly multimorbid patients with polymedication in the outpatient primary care setting,  2) to explain influencing factors on prescription of PIM, 3) to examine knowledge and application of PRISCUS and  4) to understand FPs’ reasons for prescription of PIM. |
| Waller et al.^209^ | 2005 | United Kingdom | Rational prescribing: the principles of drug selection and assessment of efficacy | Opinion piece/ commentary | n/a | To provide an overview of rational prescribing |
| Weir et al.^216^ | 2018 | Australia | Decision-Making Preferences and Deprescribing: Perspectives of Older Adults and Companions About Their Medicines | Qualitative interview study | 30 older people (aged 75+ years, taking multiple medicines) and 15 companions | To explore decision-making about polypharmacy with older adults and their companions. |
| Wilchesky et al.^210^ | 2018 | Canada | The OptimaMed intervention to reduce inappropriate medications in nursing home residents with severe dementia: results from a quasi-experimental feasibility pilot study | quasi-experimental feasibility pilot study | 44 participating residents aged 65 years or over with severe dementia was carried out in three NH in Quebec City, Canada. | To test the feasibility of an interdisciplinary knowledge  exchange (KE) intervention using a medication review guidance tool categorizing medications as either “generally”, “sometimes” or “exceptionally” appropriate for NH residents with severe dementia. |
| Williams et al.^211^ | 2004 | USA | The Short-Term Effect of Interdisciplinary Medication Review on Function and Cost in Ambulatory Elderly People | A randomized-controlled trial. | Community-dwelling older adults taking ﬁve or more medications were assessed at baseline and 6 weeks. A medication-change intervention group of 57 elders was compared with a control group of 76 elder adults. | To determine whether a medication review by a specialized team would promote regimen changes in elders taking multiple medications and to measure the effect of regimen changes on monthly cost and functioning. |
| Zechman et al.^84^ | 2019 | Switzerland | Barriers and enablers for deprescribing among older, multimorbid patients with polypharmacy: an explorative study from Switzerland | Mixed methods interview study | patients of a cluster-randomized study in Northern Switzerland. | To explore attitudes, beliefs, and concerns towards deprescribing among older, multimorbid patients with polypharmacy who chose not to pursue at least one of their GP’s offers to deprescribe. |
