# Supplementary material for: Optimising a person-centred approach to stopping medicines in older people with multimorbidity and polypharmacy using the DExTruS framework: a realist review
Source: BMC Med. 2022 Aug 31;20:297. doi: 10.1186/s12916-022-02475-1 (PMC9429627; doi:10.1186/s12916-022-02475-1)
Supplement: Supplementary file 3 — Additional file 3. CMOCs with supporting data extracts. [file 12916_2022_2475_MOESM3_ESM.docx]

## CMOCs with supporting data from included documents

| **Realist Analysis for System Level Factors** |
| --- |
| **CMOC1:** In the absence of applicable deprescribing guidelines and evidence **(C)**, healthcare providers may feel like they cannot make justifiable decisions regarding medication changes **(O)** because they don’t feel like these decisions are supported by the system **(M)**.   - *The GP quoted as follows: we can’t justify that you can just stop prescribing it. We can’t really do that because of how our treatment criteria look, where there aren’t any defined criteria for when we can stop (interview with GP18).* ***Nixon and Vendelo 2016*** - *GPs adopted a passive approach to medication management due to their uncertainty (lack of psychological capability) about which medications were most valuable in patients with multimorbidity, especially given the absence of satisfactory guidelines in this field.* ***Sinnott et al. 2015*** - *GPs generally felt insufficiently supported by the guidelines in their efforts to treat hypertension in older people… I would really like to have a guideline that states: in elderly you have pay attention to this, this and this. (GP 9, female, 5–10 years’ experience)* ***van*** ***Middelaar et al. 2018*** - *For policymakers interested in promoting discontinuation of medication, our findings suggest that attempting to de-stigmatise discontinuation is likely to benefit that interest. In the pursuit of that option, guidelines for prescription of medication must systematically describe discontinuation of medication as a possibility.* ***Nixon and Vendelo 2016*** - *Despite these misgivings, in the absence of alternative evidence, many clinical guidelines become widely used and prescribers feel under pressure to adhere to them instead of prioritising the medical treatment and deprescribing where appropriate.* ***Cullinan et al. 2017*** - *I think specifically, where we are now, with not being fully qualified doctors yet, I think you’re generally a bit more concerned about missing something or making a mistake, so you’d better do what it says in all the recommendations to protect yourself (interview with GP19).* ***Nixon and Vendelo, 2016***   **CMOC2:** When healthcare providers feel like they cannot make justifiable decisions that are supported by guidelines **(C)** they may be reluctant to make changes to medications **(O)** because they are afraid of negative consequences **(M).**   - *Physicians reported comfort in deprescribing preventive medication, but fewer were comfortable with deprescribing guideline- recommended therapeutic medications in patients with poor life expectancy. One explanation may be the fear of adverse withdrawal effects, which were also mentioned by physicians as a potential factor that prevented them from deprescribing.* ***Djatche et al. 2018*** - *A medico-legal angle on deprescribing was broached by both GPs and* *pharmacists. GPs discussed exercising caution with initiating medication changes.* **Palagyi et al. 2016** - *Perhaps the most stark identified barrier was the theme of ‘fear’– of being castigated by other professionals for not following guidelines, of the medicolegal implications of this way of working, and a lack of confidence in making and recording “defendable decisions”.* ***Reeve et al. 2018*** - ***“****We can’t justify that you can just stop [prescribing] it. We can’t really do that because of how our treatment criteria look, where there aren’t any defined criteria for when we can stop (GP18).”* ***Nixon and Vendelo, 2016*** - *The Australian GPs were overwhelmingly negative about aged care and expressed dissatisfaction at the financial reimbursement provided for ACF services. Their attitudes towards deprescribing for ACF residents were influenced by concerns of blame in the case of negative health outcomes.* ***Bolmsjo et al. 2016***   **CMOC3:** When healthcare practitioners are not supported by incentive and policy structures **(C)** they may not be able to take the time necessary for complex medication management processes **(O)** and be reluctant to make changes **(O)** because they don’t feel supported to do so **(M)**.   - *Participants discussed the challenges of demonstrating the impact of individually tailored care beyond the effect on an individual patient –“will be difficult to quantify downstream costs that will be saved - mainly by those outside of my practice so I will do work and savings will be made elsewhere* ***Reeve et al. 2018*** - *Patients who are most in need of a home medication review and most complex were considered less likely to receive equally detailed reviews because pharmacists seemed unwilling to substantially extend the review duration without additional remuneration.*  ***Mc Namara et al. 2017*** - *Inability to maintain follow-up to support a gradual process of deprescribing was a major frustration. One participant cited administrative rules as an impediment to follow-up: I think that the 2-year window for reimbursement makes it difficult to follow-up, especially for the complex patients that need that stepwise approach (CP)* ***Anderson et al. 2017***   **CMOC4:**  When healthcare professionals cannot access information about a patient’s medication regimen **(C)** they do not have an accurate understanding of the medication regimen **(O)** because they don’t understand the patient’s history **(M).**   - *Optimizing a patient’s medication is predicated on having an accurate understanding of the medications that patient is taking. This can be easier said than done, with multiple health providers, transitions of care, and potential use of over-the-counter drugs.* ***Ouellett et al. 2018*** - *GPs found that there were discrepancies between the systems across sectors and that they were not properly informed about the changes made at the hospital... “Often we are not informed about the changes. It is us, the GPs, that must try and figure it all out, that isn’t easy.”* ***Laursen 2018*** - *There were mixed views on the quality and extent of documentation of medicines in patient records… “ The problem, many times there is no documentation about the medication. Most people write documentation. Some don’t write it. Some write incoherent handwriting.” (Physician Z2, Internist)* ***Al Shemeili et al. 2016*** - *Participants were frustrated about the lack of a centralised and universal database that allowed timely access to client’s health and medication information. Home care nurses highlighted this information is important: Nobody can access the same file for every client…There’s the need for the client's chart to be accessible.* ***Sun et al. 2019*** - *All groups discussed health system structure concerns including limited funding and incomplete medical and medication histories. Medication histories rarely detail why and when medications were initiated and which prescriber is responsible for ongoing monitoring.*  ***Turner et al. 2016***   **CMOC5:** When healthcare professionals are unsure about who’s medication management is **(C)** they may struggle to engage in making medication changes **(O)** because they don’t feel they have ownership over the process **(M).**   - *Some physicians were of the opinion that they dealt with the management and control of their specialist condition only, and while this may involve an element of polypharmacy in the use of several medicines, they considered polypharmacy to be the responsibility of others.* **Al Shemeilie et al. 2016** - *Whilst an oncologist may be in a good position to judge prognosis and whether a medication is appropriate, he or she may defer to a patient’s GP regarding such medications which are usually initiated in primary care. Thus neither party may feel that they have ownership of the problem.* **Cashman et al. 2010** - *GPs were less certain that other prescribers respected their medication management roles, including deprescribing decisions, and believed that communication with other prescribers was poor…. The continued prescribing of potentially inappropriate medications is likely in a system that has a lack of clarity around roles, responsibilities and paths of communication* ***Gillespie et al. 2018*** - *The current results revealed disparities in opinion regarding the assignment of medication management responsibilities. While the GP suggested palliative care specialists were ultimately responsible for medication optimisation in CPC patients, the palliative care specialists suggested a need for increased GP involvement in medication optimisation and regimen simplification*. ***Kuruvilla et al. 2018*** - *The literature abounds with studies illustrating this confusion over where responsibility for deprescribing lies*. **Cullinan et al. 2017** - *Problems evolve because nobody assumes responsibility for optimising care-plans and because of poor coordination. Many participants concluded that their current situation did not allow them to shoulder these responsibilities. Only GPs asserted themselves as being key care coordinators for patients.* ***Mc Namara et al. 2017*** |

| **Realist Analysis for Healthcare Professional Level Factors** |
| --- |
| **CMOC6:** When a healthcare professional has previous experience deprescribing medication **(C)** they are more likely to feel able to deprescribe **(O)** because they know what to do and expect **(M)**.   - *Both sets of GPs expressed confidence in their ability to deprescribe. “After 20 years I know exactly what I want to do. I don’t have a problem with saying, yep, the Statin goes, etc.” (AusGP4)* ***Bolmsjo et al. 2016*** - *Compared to younger GPs, older GPs were signiﬁcantly more likely to agree they were conﬁdent to deviate from clinical guidelines (42.9% v 72.7%; P= 0.030).* ***Gillespie et al. 2018*** - *Later on now in my career, I’ve taken on a different approach. I understand that managing polypharmacy is an art as much as it is a science. You have to balance quality of life, risks and beneﬁts, when prescribing medications. I don’t feel the need to ﬁx everything.* ***Hernandez 2017*** - *Experience might also contribute to self-confidence and how risk is viewed. Negative experiences reinforced a tendency to opt for the status quo, whereas positive or neutral experiences fostered open-mindedness toward deprescribing.* ***Anderson et al. 2017*** - *Best guesses were also required because ‘you don’t have guidelines for every situation — there are times when you just have to make a decision as best you can’ (GP6). GPs relied heavily on their prior knowledge and experience of the patient in this process.* ***Sinnott et al. 2015***   **CMOC7:** When healthcare professionals feel they don’t have the necessary skills and knowledge  to manage medicines in older adults **(C)** they are less likely to make changes to patients’ medicine regimes **(O)** because they are not confident in their ability to make good decisions **(M)**.   - *“I’m not clever enough to have all the statistics in my head to be able to say, well, that Statin is stopping all that absolute relative blah de blah, which I don’t understand very well. So I can’t really educate the patient off the top of my head.” (AusGP4)* ***Bolmsjo et al. 2016*** - *Often, the GPs needed to discuss the patient’s treatment because they did not feel they had the knowledge or skills to make correct therapeutic decisions. As one GP stated, “A specialized treatment belongs at the hospital, where the specialist can use their expertise.”* ***Laursen et al. 2018*** - *Physicians, nurses and pharmacists proposed that there was an inherent need for specialised education and training in medicines management for elderly patients. You need to have training, because like paediatrics, geriatric population needs speciﬁc involvement. Even the pharmacokinetics is different like the paediatrics. (Physician A2, ICU)* ***Al Shemeili et al. 2016***   **CMOC8:** When medicines have been prescribed by a specialist **(C)** other healthcare provider from other specialities may be reluctant to make changes to patients’ medicine regimens **(O)** because they do not feel they have the knowledge to make a safe decision **(M**).   - *Often they will stop these days and just go back to one so I would question the dipyridamole but not necessarily stop it. Looks like the cardiologist has prescribed dipyridamole so I guess we would accept that (GP6).* ***Ailabouni et al. 2016*** - *When asked about barriers to medication withdrawal, the GPs felt that … it was their job to ensure the medication list was as accurate and up to date as possible. … GPs felt it was difficult to discontinue medication that a specialist at the hospital had prescribed. .”* ***Laursen et al. 2018*** - *Prescription by a specialist … is a factor … that inhibits many FPs from deprescribing medications and has been noted previously … this reluctance may be due to lack of confidence in their own deprescribing knowledge and experience or being unclear about the indication for the specific medication.* ***Harriman et al. 2014***   **CMOC9:** When medicines have been prescribed by another healthcare professional **(C),** healthcare providers may be reluctant to make changes to patients’ medicines **(O)** because they are worried about damaging relationships with the original prescriber as well as between the original prescriber and the patient **(M).**   - *There was also a reluctance to ‘interfere’ with other healthcare providers’ prescribing driven by fear of disturbing therapeutic relationships, hesitation to contradict prescribing by other healthcare providers.* ***McNamara et al. 2017*** - *GPs were reluctant to discontinue medication prescribed by other medical specialists without contacting them. Contacting the specialist to change medication, however, took additional effort and GPs feared that it would be difficult to reach a consensus as the specialists often have a different viewpoint.* ***Rieckert et al. 2018*** - *The participants described a perceived ‘hierarchy of prescribers’ meaning that GPs may have inhibitions to question prescriptions from clinicians or specialists. The professor said yesterday what you have to take in the afternoon and now the little GP comes and says: You don’t have to take all that. Then the question is, whether the GP or the professor is right (Focus group 2012)* ***Straßner et al. 2018*** - *Furthermore, GPs reported complexities communicating with specialist physicians. GPs discussed being intimidated by specialist physicians for deprescribing medications they initiated, with one recounting being ‘scorned by a colleague’. Furthermore, GPs expressed disappointment when deprescribed medications were restarted by a specialist physician or in hospital.* ***Turner et al. 2016***   **CMOC10:** When healthcare professionals don’t have dedicated time **(C)** they may be less likely to make changes to patients’ medications **(O)** because they do not have the emotional and cognitive capacity to consider complex issues **(M).**   - *It is important to consider a patient’s goals of care, prognosis, and functional level when considering which medications are potentially inappropriate. The process of deprescribing also requires special attention and time.* ***Pruskowski and Handler 2017*** - *Participants also emphasized…that communication should go beyond checks on understanding of information to communicating the benefits of any changes and being responsive to patient concerns. Both patients and professionals agreed this would require dedicated, protected time to enable issues to be explored.* ***Knowles et al. 2017*** - *GPs currently servicing Long-term-care facilities (TCF) were overwhelmed with day-to-day responsibilities, less likely to engage in time-consuming tasks such as medication reviews and deprescribing decisions, and generally preferring to maintain the status quo.* ***Palagyi et al. 2016***      - *Consumers felt relatively uncomfortable speaking about medications during medical consultations because of lack of time to discuss issues with physicians.* ***Manias et al. 2017*** - *lack of time pushed GPs towards ‘maintaining the status quo’, …especially if considering changing ‘something that you have been giving them for the last 15 years — … you’re suddenly saying the evidence is saying that we shouldn’t be giving you aspirin anymore — it takes time, time to explain that’.* ***Sinnott et al. 2015*** - *Prescribing HCPs, particularly GPs, described how the limited time available for care episodes reduced the likelihood of incorporating patients’ preferences into treatment decision-making for multimorbidity.* ***Mc Namara et al. 2017***   **CMOC11:** When healthcare professionals do not have time (C) they may find it difficult to fully consider a patient’s care goals **(O)** because they are forced to prioritise what they spend their time on **(M).**   - *The monitoring and follow-up stage is usually the most time-consuming aspect of the process…. Deprescribing goes beyond identifying a list of medicines to be stopped, and there should be sufﬁcient time built into the process to allow adequate monitoring and follow-up.* ***Oboh et al. 2018*** - *The process of discontinuing a medication is also time consuming; the physician must critically review a patient’s medications, meet with the patient’s family, explain and dispel misconceptions about deprescribing, and slowly taper and discontinue medications while following the effects of these changes over several weeks or months.* ***Harriman et al. 2014*** - *Key barriers to engagement related to the practical constraints of workload placing limits on the necessary time and ‘head space’ needed to engage with this complex form of clinical practice. “limited by time, caseload and so lack of mental capacity” (GP) “I barely get through the day reacting” (GP)* ***Reeve et al. 2018*** - *Another limitation is the intensive nature of the intervention itself. As implemented in this pilot study, the intervention requires a significant amount of time (estimated 2–3 h per patient).* ***Petersen et al. 2018*** - *Participants expressed their concerns about their workload and how their overwhelming work schedule leaves little room for medication review and reconciliation. One participant shared that time constraint discouraged nurses from engaging in a complete medication review and reconciliation process with their clients at home.* ***Sun et al. 2019*** |

| **Realist Analysis for Patient Level Factors** |
| --- |
| **CMOC12**: When patients believe medicines are a sign of good care (C) doctors may be reluctant to consider deprescribing **(O)** because explaining and justifying any deprescribing is more emotionally and cognitively taxing (**M**).   - *It shatters basic almost ‘religious’ worldwide beliefs that we all perceive from childhood regarding health–disease and patient–caregiver interactions. The good doctor is supposed to heal but throughout history, the patient–doctor interaction is increasingly perceived as a disease–drug interaction.* ***Garfinkel 2017*** - *Patient expectations also drive polypharmacy. With an increasing focus on the provider to take action to increase patient satisfaction, prescribing is seen by patients as a sign of caring.* ***Chen and Buonano 2017*** - *First, patients and providers alike possess psychological connections to medications. This may be because medications are the most visible form of health care.* ***Pruskowski and Handler 2017*** - *Moreover, in the absence of “low-hanging fruit” or a clear trigger to cease therapy, deprescribing, compared with initiating therapy, appears a riskier, less certain, and more cognitively and socially demanding process, with minimal decision support.* ***Anderson et al****.* ***2017***   **CMOC13:** When patients believe their medicines are providing them with benefits **(C)** doctors may find it difficult to discuss deprescribing (**O)** because explaining and justifying any deprescribing is more emotionally taxing **(M).**   - *GPs had specific difficulties talking to multimorbid patients about stopping medications; they feared this could be interpreted by the patient as a withdrawal of care and potentially damage the doctor– patient relationship:’* ***Sinnott et al. 2015*** - *Factors influencing GPs’ deprescribing were beliefs concerning patients (patients have no problem with polypharmacy; patients may interpret a proposal to stop preventive medication as a sign of having been given up on; and confronting the patient with a discussion of life expectancy vs quality of life is ‘not done’* ***Schuling et al. 2012*** - *GPs acknowledged that in the terminal phase it would be rational to discontinue anti-hypertensive-medication. However, they often hesitated to take this step to avoid the impression they were giving up on the patient or unnecessarily deprive them of a sense of being in control with adequate BP measurements.* ***van*** ***Midelaar et al. 2018***   **CMOC14:** When patients believe a medicine might be working or will work in the future **(C)** they are likely to want to continue taking it **(O)** because they hope they are doing something to help their condition **(M).**   - *Patients feel an improvement when they start taking the drug or hope for a future improvement and for that reason they do not want to discontinue the drug. Some … think that they are doing something that can help their condition and in doing so they feel reassured.* ***Goncalves 2018*** - *Patients and family members sometimes cling to the hope of future effectiveness of a treatment.* ***McGrath et al. 2017***   **CMOC15:** When patients believe their medicines as keeping them alive **(C)** healthcare professionals may find it difficult to discuss deprescribing **(O)** because they don’t want their patients to feel they have abandoned them **(M).**   - *Moreover, GPs are reluctant to initiate a discussion about stopping medication because they are concerned that patients may interpret this as a sign of being given up on. – People may then get the feeling, ‘Don’t I count anymore, am I not important?’* ***Schuling et al. 2012*** - *They also considered the reaction of the patients, who might have come to value their medicines or feel that deprescribing is a sign of abandonment.* ***Bokhoff and Walker 2016*** - *Also important when discussing goals is to anticipate …and discuss any barriers with patients and carers. These may include patient-based barriers, e.g. psychological discomfort when ceasing a medication they have been taking for many years, or feeling their situation is hopeless since medications for chronic diseases are being ceased.* ***Hardy and Hilmer 2011*** - *For deprescribing to be successful, both healthcare professionals and patients should be actively engaged in it. Studies have shown that some general practitioners find it challenging to deprescribe medications in elderly patients with multimorbidity, believing patients could perceive it as a sign of being given up on.* ***Akinbolade et al. 2016***   **CMOC16:** When patients view medicines as prolonging their lives **(C)** they may be reluctant to stop taking them **(O)** because they view deprescribing as a sign that they aren’t worth keeping alive anymore (**M).**   - *Moreover, GPs are reluctant to initiate a discussion about stopping medication because they are concerned that patients may interpret this as a sign of being given up on. – People may then get the feeling, ‘Don’t I count anymore, am I not important?’* ***Schuling et al. 2012*** - *Still other physicians voice concerns that patients will feel the physician is “giving up on them” or “leading them to quicker deaths.”* ***Harriman et al. 2014*** - A number of residents expressed fear at the concept of reducing or ceasing some of their current medications, believing that their medicines were prolonging their life. ***Palagyi et al. 2016*** - This participant explained that … her life must not be burdensome to others: “If I were to stop all the medications, I’d be a vegetable or dead. I don’t want to be a burden to my family, I want to live as long as I have quality of life.” ***Ross and Gillett 2020***   **CMOC17**: When patients believe medicines are providing them with benefits **(C)** patients may be reluctant to discontinue them **(O)** because they are afraid of negative consequences **(M).**   - *However, drug use could equally represent a threat to identity. Thus drugs could both be seen to restore previous identities threatened by chronic illness and to be the concrete representation of the threat to, or loss of, that identity.* ***Townsend et al. 2003*** - *The overarching pattern of “preserving self” was a surprising and clear ﬁnding. Taking medication was closely tied to self-identity and manifested in various ways, described in the ensuing sections. Taking multiple medications was signiﬁcant and personal.* ***Vandermause et al. 2016*** - *One of the stories often told is that “medications keep you healthy.”…,suggesting that it is through medications that aging is optimized. Medications are seen as a possible means of mitigating the common fears associated with aging, including a lack of independence, immobility, and deterioration in cognitive function.* ***Ross and Gillett 2020*** - *Mrs. D derives an important sense of empowerment from her supplement use that should be respected by her physicians.* ***Pitkala et al 2016*** - *Accounts revealed how involvement constituted a function of participants’ conceptualisation and presentations of self, as well as how individuals believed they were seen by others in their networks, i.e. their identities or roles within their networks.* ***Cheraghi-Sohi et al. 2015*** - *Participants valued their medicines because they are a necessary part of life and most people they know are taking them. Medicines also “work” by keeping them alive, reducing symptoms such as pain, and improving quality of life.* ***Weir et al. 2018*** - *“I would love to reduce medication. Nevertheless, I need the drugs my GP wanted to stop.” P3 “I have good experience with my drugs…, so better not change the winning team.” P12 “I feel well now, obviously all of my drugs are needed.” P15* ***Zechman et al. 2019***   **CMOC18:** When families or carers perceive medicines to have a benefit for the patient **(C)** healthcare professionals may be reluctant to consider deprescribing **(O)** because they feel pressured not to do so (**M**).   - *Several GPs talked about the challenge of keeping patients on potentially unnecessary medication at the urging of family members.* ***Jansen et al. 2017*** - *GPs discussed exercising caution with initiating medication changes, particularly where they assumed a resident’s family had strong expectations of medicines keeping their relative alive. “I think we really need to be … educating relatives about what is realistic because it’s very hard to initiate that discussion. (GP; female 59 years)* ***Palagyi et al. 2016*** - *Ms L: The family is all guilt-ridden and ..tell themselves that they have to keep dear dad alive …. Like many older patients in the final chapter of their lives, Mr L faces choices about using medications that may increase his longevity but negatively affect his quality of life.* ***Steinman and Hanlon 2010***   **CMOC19:** When families/carers are involved in a patient’s healthcare **(C)** patients may be more able to engage in decision-making about their medicines **(O)** because they feel supported by them **(M).**   - *This patient mentioned relatives as an important means of support. …Her daughters look up information for her. She discusses her disease and treatment with her daughters, who motivate her to go to the doctor and address difﬁcult topics. The daughters sometimes accompany her, so they are informed and ask questions.* ***Schopf et al. 2018*** - *Families can facilitate exchange of information and encourage patient engagement in their healthcare. Studies have found that ofﬁce visits in which the older adult patient was accompanied by a companion who prompted their involvement were 4.5 times more likely to be involved in decision making than their counterparts.* ***Hernandez 2017*** - *Medication-taking appears to be a personalised, contingent and contextually situated type of work with participants developing highly individualised routines and strategies. However, it was also evident that daily routine medication-work can and does go beyond the self, with network members regularly supporting … particular type(s) of work.* ***Cheraghi-Sohi et al. 2015*** |

| **Realist Analysis for Shared Decision Making** |
| --- |
| **CMOC20:** When healthcare professionals involve patients in the medication management process **(C)** they are more likely to make better decisions about medication **(O)**, because of their shared expertise **(M)**.   - *For older adults, decades of observing their physical reactions to various medicines and other dietary behaviours locates them as experts of their own bodies. Health care teams must recognize this knowledge if deprescribing programs are to become standard medical care for older adults.* ***Ross and Gillett 2020*** - *The stories of patients and caregivers, their representations, perceptions, experiences and preferences can reduce the risks of inappropriate exams and treatments. …. Using the patients' and caregivers' stories, the interdisciplinary teams can interact better, focussing not on the single pathology, but on the individual as a complex system.* ***Cenci 2016*** - *Establishing the importance of symptoms and outcomes with patients and carers; … will help guide deprescribing decisions… Also anticipating barriers to deprescribing … such as psychological discomfort when ceasing a medication, or feeling their situation is hopeless since medications for chronic diseases are being ceased.* ***Hardy and Hilmer 2011*** - *… some GPs …were particularly good at tailoring to the older patient. They were more aware of evidence around CVD risk management in older adults, were concerned about balancing harms/benefits of preventive medications, recognised lifestyle change is achievable for older adults, …and engaged older adults and carers in decision making.* ***Jansen et al. 2017*** - *We propose that an individualised structured medication review, where deprescribing is considered alongside prescribing, and where there is active engagement with patients on their views on treatment is essential to tackling polypharmacy and inappropriate prescribing.* ***McCarthy et al. 2017*** - *Embedding shared decision making… may be a challenge; but it is one that will differentiate a HMR as a valued service. By exploring the patient’s beliefs and attitudes the conversation may be more likely to result in genuine agreement, with the patient taking the medicine as prescribed.* ***Rigby 2013*** - *The aim of the medication assessment is to collect information about… medication use and to gain an understanding of the patient and his/her wishes, experiences, and beliefs about medications… enabling the doctor to make rational decisions about medications and to determine whether the patient's medication‐related needs are being met.* ***Drenth-van Maanen et al. 2017***   **CMOC21:** When healthcare professionals are aware of a patient’s perspectives and beliefs about medicines and their goals of care **(C)** they are more likely to achieve patient-centred outcomes **(O)** because the patient is understood **(M)**.   - *A key component of addressing the risks associated with polypharmacy is to ensure that patients are fully involved in the decision to start a drug; and also in monitoring their use of medication to ensure appropriate adherence to their prescribed drug regime.* ***Molokhia and Majeed 2017*** - *We conclude that a high level of patient involvement and SDM does not necessarily mean that patients will pursue deprescribing when offered... A joint decision to continue a medication rather deprescribing could be the best decision for the patient if in line with her/his values and preferences.* ***Zechman et al. 2019*** - *Deprescribing should be carried out with the agreement of the patient and carers. They should be informed of the purpose of the medications and the reasons for withdrawing some of them.* ***Goncalves, 2018*** - Perhaps the minimum requirement for shared decision making in this context is establishing awareness of the option to be involved; discussing preferences over time, as they may change; and, if a person is interested in being involved, creating the circumstances for this. ***Weir et al. 2018*** - *Establishing the importance of symptoms and outcomes with patients and carers will help guide deprescribing decisions. Such discussions may require decisions about relaxing targets for therapy, such as levels for blood pressure or blood sugar and whether to perform pathology tests.* ***Hardy and Hilmer 2011***   **CMOC22:** When healthcare professionals involve patients in the decision-making process **(C)** they are more likely to make defendable decisions about medications **(O)**, because of their shared responsibility **(M)**.   - *It is important to respect and acknowledge the patient’s choice in the use of medicines and to ensure that the risks and benefits of their decisions are shared and documented.* ***Kaufman et al. 2017*** - *A patient’s attitudes to change could relieve the clinician of responsibility for deprescribing: The GP doesn’t necessarily have to provide the answer. It will be the patient who will provide the answer as to how willing he or she is to stop something (GP).* ***Anderson et al. 2017*** - *Our interviews suggest that few GPs use a shared decision-making approach and many refer to challenges associated with older patient involvement… Whereas a focus group study on secondary CVD prevention in which the majority of GPs mentioned using shared decision making as a way to deal with uncertainties….* ***Jansen et al. 2017*** - *This contrasted with cases in which the GP shared the uncertainty and responsibility for a decision with the patient… You have to go “this is your life, your decision” and then give them my advice but they have to make the decision for themselves.’ (GP3 discussing primary prevention)* ***Sinnott et al. 2015*** |
|  |
| **Realist Analysis for Continuity of Care and Building Trust** |
| **CMOC23:** When patients are presented with conflicting recommendations about their medication by health care professionals **(C)**, their trust may decrease **(O)**, because they don’t know who to believe **(M)**.   - *Participants emphasised that there is a need for continuity of care to support safe deprescribing. Otherwise, different healthcare providers might have different pieces of advice for their clients, then it would be difficult to build a therapeutic relationship between the care providers and care recipients.* ***Sun et al. 2019*** - *Consistency not redundancy among healthcare providers is important. When doctors, pharmacists and nurses are all telling the same story… then this should go over a lot better… we must send a consistent message, not a conflicting message. (FG2, P3)* ***Sun et al. 2019*** - *Further patients distrusted the healthcare system after having poor experiences such as no follow-up, not being heard, or conﬂicting advice given by different doctors...* ***Bokhof and Walker 2016*** - *The second reason was a more general distrust of the complex healthcare system and its various stakeholders. It was repeatedly seen as a problem that consulting several doctors (GPs and specialists) frequently led to uncoordinated prescription of multiple different medicines.* ***Uhl et al. 2018***   **CMOC24**: When patients and their carer/family are asked to change their usual medication by a health care professional they are unfamiliar with **(C)**, they may be reluctant **(O)**, because they are concerned the person does not know what is best for them personally **(M)**.   - *The second highest ranked factor by residents, ‘continuity of nursing staff’, related to communication with known and trusted health professionals. Residents commented that unfamiliar nurses were unlikely to be aware of their medical, social and medication history and preferences – …. This was a perceived as barrier to deprescribing.* ***Turner et al. 2016*** - *Unfamiliarity with the medical team during hospitalisation may lead to resistance among patients and family members when deprescribing.* ***Nadarajan et al. 2018***   **CMOC25:** When a health care professional demonstrates to a patient they understands their needs and goals **(C)** the patient is more likely to trust them **(O)** because they believe the heath care professional is acting in their best interest **(M)**.   - *The reasons participants gave for this were trust, a long relationship, and a belief that their doctor is aware of their preferences. “Well, um, he knows best. He knows my condition. …. So he knows me inside and out sort of thing” (ID21, female, age 80)* ***Weir et al. 2019*** - *This study alludes to the importance of establishing trust in the physician as a pre-requisite … to effectively influence change in attitudes and practices of patients towards optimal health outcomes. …Physician trust can be enhanced via continuity of care…, physician personality … and patients’ perceived freedom to select choice.* ***Ng et al. 2017*** - *A physician can counsel the patient as to why a medication is not indicated …. However, this counselling often requires the development of trust, and the patient having a sense that the physician understands what he or she feels… that the physician has their best interest in mind.* ***Chen and Buonanon 2017***   **CMOC26:** When a patient trusts their healthcare provider **(C)** they may be more likely to consider changes to their medication **(O)** because they believe their healthcare professional is acting in their best interest **(M)**.   - *Clinicians must recognise that the client-health provider relationship is critical to prevention of polypharmacy. Within the context of caring, collaborative relationships, the clients are more likely to respond openly to questions about factors influencing their patterns of medication usage including cost, complexity and lifestyle.* ***Jones, 1997*** - *Patients who had higher trust scores in their physicians were more likely to be in favour of deprescribing* ***Ng et al. 2017*** - *Considerable scholarly work has previously examined the operation of trust in health-care encounters, maintaining that trust is necessary amidst uncertainty, vulnerability and particularly for older adults. When studied against the backdrop of polypharmacy and deprescribing, trust remains an essential ingredient in the health-care needs of … older adults.* ***Ross and Gillett, 2020*** - *Trust matters if the older adult placed on a deprescribing plan is vulnerable to future consequences. It could be the case that remaining on certain medications may lead to problematic outcomes; or perhaps discontinuing certain medications that the older adult believes are vital may cause significant uncertainty and stress).* ***Ross and Gillett 2020***   **CMOC27:** When healthcare professionals know that they will be able to follow-up a patient **(C)**, they are more likely to try deprescribing **(O)**, because they are reassured they will be able to manage potential harms **(M).**   - *For GP participants, a continuous therapeutic relationship with a patient was critical to better assessing harms and benefits and committing to the potentially protracted process of deprescribing: That’s the starting point: who the person’s primary GP is.* (GP). ***Anderson et al. 2017*** - *The process of discontinuing a medication is also time consuming; the physician must critically review a patient’s medications, meet with the patient’s family, explain and dispel misconceptions about deprescribing, and slowly taper and discontinue medications while following the effects of these changes over several weeks or months.* ***Harriman, 2014*** |

| **Realist Analysis for Monitoring** |
| --- |
| **CMOC28:** When a clinician judges that a patient may benefit from a change in medication **(C),** they are likely make small incremental changes **(O)** because they are concerned about causing harm to the patient **(M).**   - *If you are going to taper a medication, develop a schedule in partnership with the patient. Stop one medication at a time so that you can monitor for withdrawal symptoms or for the return of a condition.* ***McGrath et al. 2017*** - *The strategies ‘Start low, go slow’ and ‘Trial and error’ could be considered if the physicians were uncertain about prescribing drugs to older patients and acted according to this uncertainty.* ***Christensen et al. 2017*** - *Once a discontinuation regimen has been decided, selected drugs can then be ceased or weaned, one at a time, while monitoring the patient closely for disease recrudescence or onset of withdrawal or rebound syndromes...* ***Scott et al. 2013*** - *Participants emphasised that finding the right timing to deprescribe inappropriate and unnecessary medications is the essence of successful deprescribing. Home care nurses added that removing inappropriate and unnecessary medications require a proper schedule of tapering off medication dosages gradually over a period of time.* ***Sun et al. 2018***   **CMOC29:** When a harms minimisation process is provided by clinicians during medication changes **(C)**, patients are more willing to make these changes **(O)**, because they feel reassured **(M).**   - *As the patient was not experiencing regular nocturnal leg cramps, the ICP suggested stopping the quinine. The patient was agreeable, but he wanted to keep some at home, just in case.* ***Oboh and Qadir, 2016*** - *Finally, the thought of medication discontinuation can evoke fear in some patients, even with medications not commonly associated with withdrawal reactions. Patients report feeling …more willing to trial cessation if the medication is reduced gradually. However, the most effective tapering regimens are mostly unknown.* ***Reeve et al. 2014*** - *Close monitoring of, and discussion with, the patient throughout the period of medication change will determine whether goals are being met, detect any adverse events, and demonstrate to the patient that the change is part of an active treatment plan.* ***Hilmer et al. 2012***   **CMOC30:** When a patient provides feedback to a clinician about the effects of a medication change **(C),** they are make an informed decision about its value **(O)**, because of their new knowledge **(M).**   - *At times, discontinued medication had to be restarted. However, in these cases the GPs were glad to have tried the discontinuation because then the decision to prescribe the medication was made more consciously and the necessity of the medication was confirmed.* ***Rieckert et al. 2018*** - *The importance of reviewing patients to monitor the effects of any changes made to prescribing are also emphasised, which would include the need to whether any further changes to treatments are needed (including restarting a treatment).* ***Molokhia and Majeed 2017*** - *Another reason why proposed medication changes did not result in observed medication changes could lie in the process of cessation. The GP and patient may have agreed to stop the medication, including a reconsideration of this decision after a specific period. This may have led to restarting the medication.* ***van Summeren et al. 2017***   **CMOC31:** When healthcare professionals are aware of a patient’s current perspective and beliefs about their medication **(C)**, patients are more likely to consider medication change **(O)** because they feel understood **(M)**.   - *Perhaps the minimum requirement for shared decision making in this context is establishing awareness of the option to be involved; discussing preferences over time, as they may change; and, if a person is interested in being involved, creating the circumstances for this.* **Weir et al. 2018** - *Preferences are not stable and can change over time and should therefore never simply be assumed; Type 1 or Type 3 participants, if provided with appropriate support, may come to value information about their medicines and desire a more active role in decision-making.* **Weir et al. 2018** - *Monitoring and follow-up after the review ensured that the changes led to the desired outcomes and minimised the risks of adverse events.* **Oboh and Qadir, 2017** - *Frequent review and amendments to goals of care are necessary when managing medical comorbidities. The need for certain medications, as well as their route and time of administration, should be evaluated regularly…..* *The entire process is meant to be iterative; symptoms, clinical trajectories, and life circumstances often change over time and may result in changes in patients’ priorities.* **O’Brien, 2015** - *Return consultations were an opportunity to re-evaluate the patient, thereby reassuring the GP and patient, giving clarity on the best approach to take, and facilitating the management of multiple competing demands.* **Sinnott et al. 2015** |

| **Realist Analysis for Multidisciplinary Collaboration** |
| --- |
| **CMOC 32:** When healthcare professionals can draw on the skills and expertise of colleagues **(C)** they feel more confident in making prescription changes **(O)** because they feel re-assured that they are making safe and optimal prescribing decisions **(M).**   - *Overall, two main options for support were valued. The first concerned meetings* *with GPs or pharmacists to discuss patients with complex problems, as a check of their expertise, and to exchange ideas and information around medication management decision making…‘* ***Sinnige et al. 2016*** - *A general standpoint was that the GPs, as generalists, did not feel they had the necessary knowledge or backup from hospital-based specialists to conduct critical medication reviews, and this highlighted the need for better cross-sectoral collaboration as well as greater education. .* ***Laursen et al. 2018*** - *The GPs were enthusiastic, eager to hear about the considerations made by their peers, and the meeting was considered useful: ‘It turns out (again) that we should discuss these patients not on our own, but in a team, as it yields more information than you anticipate.’ GP5* ***Sinnige et al. 2016*** - *General practitioners are best able to understand the complete medical, functional and social issues that are in play when optimising medications. A multidisciplinary approach involving doctors (GPs and specialists), pharmacists and nurses, is the common feature of successful interventions to reduce polypharmacy.* ***Hilmer et al. 2012*** - *Since there is little evidence or guidance in reducing or discontinuing medications for chronic illnesses during EOL care, it is important that healthcare professionals collaborate to determine which drugs are medically necessary.* ***Cimmino and Pisano 2016***   **CMOC33:** When healthcare professionals can discuss complex cases with colleagues **(C)** they feel more confident about making medication changes **(O)** because they feel supported **(M).**   - *Peer support was the key technique in generating recommendations for medication optimisation. While other professional sources (i.e. pharmacist, specialist) were reported to be useful, conducting the review with patients only was not: therefore, professional social support will be a compulsory component of any future iterations of the intervention.* ***Sinnott et al. 2017*** - *A useful source of support for some GPs in the interview study was their GP colleagues. These interactions occurred on an informal basis within practices and were notable for their ready accessibility and generalist nature.* ***Sinnott et al. 2015*** - *They found that ‘to bounce ideas off your colleagues just helps, even if it is just something like “what in the name of God am I going to do about this”’ (GP8). Single-handed GPs struggled in this regard using CPD meetings as a forum for discussing complicated cases with other GPs.* ***Sinnott et al. 2015*** - *A further issue raised by the interviewees, was the team support within the hospital environment. Particularly, the hospital pharmacist was considered a useful team member and a reliable resource.* ***Cullinan et al. 2014***   **CMOC34:** When healthcare professionals work collaboratively **(C)** they can improve continuity of care **(O)** and their understanding of their patients’ needs **(O)** because they can share workload **(M).**   - *Nurses’ prescribing permission differs among countries and in some of them they have no permission at all. Even so, they may inﬂuence prescribing because they observe and can communicate to physicians the treatment burden associated with polypharmacy* ***Goncalves 2018*** - *Results of the medication review were discussed within nMDT …. Residents were monitored for adverse events by care home staff …and followed up by the pharmacist to ensure suspected negative effects were identified and safely managed. The process was iterative, with rapid feedback … to improve the process.* ***Baqir et al. 2014*** - *Hospital, clinic and community nurses can play an important role in assisting patients with adherence and in clarifying the accuracy of a medication list. Collaboration with clinical pharmacists has been shown to be an important strategy to reduce inappropriate medications and to help deprescribe as appropriate.* ***Frank 2012*** - *Nursing’s biopsychosocial approach is well placed to explore patients’ health beliefs and the inﬂuence these have on medication taking behaviours. Such questions could be part of a patient admission process with regular review during an episode of care.* ***Naughton and Hayes, 2016*** |
